# Natural killer cells against viral infection: from basic biology to immunotherapy

**DOI:** 10.1093/pcmedi/pbag020

**Published:** 2026-07-14

**Authors:** Zicheng Zhang, Fangjie Wang, Rongjiao Liu, Lei Tian, Liang Cai, Youcai Deng

**Affiliations:** Department of Pharmacology and Department of Clinical Hematology, College of Pharmacy, Army Medical University, Chongqing 400038, China; State Key Laboratory of Trauma and Chemical Poisoning, Army Medical University, Chongqing 400038, China; State Key Laboratory of Trauma and Chemical Poisoning, Army Medical University, Chongqing 400038, China; The First Research Department, Army Medical Center (Daping Hospital), Army Medical University, Chongqing 400042, China; Hunan Provincial Center for Disease Control and Prevention (Hunan Academy of Preventive Medicine), Changsha 410153, China; Division of Hematology/Oncology, Department of Medicine, School of Medicine, University of California, Irvine, CA 92697, USA; Hunan Provincial Center for Disease Control and Prevention (Hunan Academy of Preventive Medicine), Changsha 410153, China; Department of Pharmacology and Department of Clinical Hematology, College of Pharmacy, Army Medical University, Chongqing 400038, China; State Key Laboratory of Trauma and Chemical Poisoning, Army Medical University, Chongqing 400038, China

**Keywords:** natural killer (NK) cells, viral infection, immunotherapy

## Abstract

Natural killer (NK) cells, a pivotal component of the innate immune system, exert indispensable roles in antiviral immunity by directly eliminating virus-infected cells and modulating adaptive immune responses. This review systematically synthesizes the biological characteristics of NK cells and their multifaceted functions against viral infections. The bidirectional crosstalk between NK cells and viruses is elucidated, with a focus on NK cell adaptive features and viral evasion strategies during specific viral infections. Furthermore, potential therapeutic targets and cutting-edge immunotherapeutic strategies due to modulation of NK cell activities are summarized, including monoclonal antibodies, chimeric antigen receptor-modified NK cells, and adjuvant therapy with Chinese herbal medicines. Recent clinical evidence and preclinical advances are integrated to provide a comprehensive framework for understanding NK cell-mediated antiviral immunity, identifying novel insights to guide the development of precise, effective immunotherapies for combating refractory viral infections.

## Introduction

**Figure 1:**
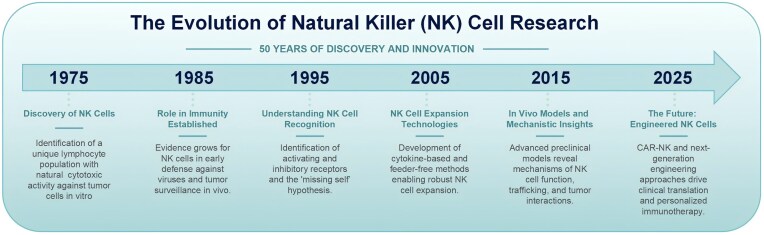
The milestones of anti-viral NK cell research. This timeline illustrates the pivotal discoveries and paradigm shifts in the field of NK cell-mediated antiviral immunity over the past 50 years. In 1975, NK cells were initially identified as a unique subset of lymphocytes capable of spontaneous, non-sensitized cytotoxicity. In 1989, a landmark clinical observation by Biron *et al*. provided the first definitive evidence of their non-redundant antiviral role in a complete NK cell-deficient patient. The fundamental mechanism of target recognition was elucidated in 1990 through the “missing-self” hypothesis, which explained how NK cells sense the downregulation of MHC-I molecules on virus-infected cells. The traditional view of NK cells as strictly innate effectors was subsequently challenged by the discovery of adaptive-like features, with antigen-driven clonal expansion and memory populations first identified during HCMV infection in humans (2004, NKG2C^+^ subset) and later confirmed in murine models (2009, Ly49H^+^ subset). In 2017, the field entered the translational era with the first clinical trial of adoptive NK cell therapy for HIV-1 infection, opening a new chapter in NK cell-based antiviral immunotherapies.

Upon viral invasion, the host immune system initiates a coordinated response to eliminate the pathogen, with the innate immune system acting within hours and the adaptive immune system within days. However, immune dysregulation can lead to death or acute-to-chronic viral transition. Among innate immune components, NK cells stand out as frontline responders, playing a pivotal role in antiviral immunity [1, 2]. The discovery of natural killer (NK) cells dates back to the mid-1970s, when they were initially characterized as a unique population of lymphocytes capable of spontaneous cytotoxicity against tumor cells and virus-infected targets without prior sensitization [3, 4]. A landmark clinical observation in 1989 by Biron *et al*. provided the first definitive evidence of their non-redundant role in human anti-viral defense, describing a patient with a complete NK cell deficiency who suffered from life-threatening herpesvirus infections [5]. Subsequently, the “missing-self” hypothesis proposed in 1990 elucidated the fundamental mechanism by which NK cells discriminate “self” from “non-self” by sensing the downregulation of major histocompatibility complex class I (MHC-I) molecules—a common strategy employed by viruses to evade T-cell recognition [6]. In the 21st century, the traditional view of NK cells as short-lived, non-specific innate effectors has been further challenged by the discovery of their adaptive-like features. Breakthrough studies on cytomegalovirus (CMV) infection in both humans in 2004 [7] and mice in 2009 [8] revealed that specific NK cell subsets (e.g. NKG2C^+^ in humans or Ly49H^+^ in mice) could undergo antigen-driven clonal expansion and persist as long-lived “memory” populations [7, 8]. The first clinical trial of adoptive NK-cell therapy for viral infection, led by Jeffrey S. Miller and Timothy W. Schacker, was registered in 2017 (NCT03346499) to evaluate haploidentical NK cells combined with IL-2 in patients with HIV-1 [9], opening a new chapter in NK cell-based antiviral immunotherapy (Fig. 1).

NK cells origin from hematopoietic stem cells (HSCs) in the bone marrow [10, 11] and exit to peripheral blood and tissues after maturation. Upon viral infection, host cells initiate the production of type I interferons (IFNs), pro-inflammatory cytokines, and chemokines, establishing a chemotactic gradient that recruits NK cells to infected tissues [1]. Concurrently, tissue-resident myeloid cells [such as macrophages and conventional dendritic cells (cDCs)] as well as plasmacytoid DCs, produce innate cytokines (including IL-12, IL-15, and IL-18) that cooperatively stimulate NK cell survival and activation [1, 2]. Once recruited and activated, NK cells directly eliminate infected cells through cytolytic granule exocytosis containing perforin and granzymes, or by initiating death receptor-mediated apoptosis via Fas ligand (FasL) and TNF-related apoptosis-inducing ligand (TRAIL) [12]. Beyond direct cytotoxicity, activated NK cells modulate adaptive immunity by participating in reciprocal cellular crosstalk and secreting immunoregulatory cytokines, primarily IFN-γ and TNF-α, which promote DC maturation and helper T-cell priming [13]. This rapid and robust innate response during early infection is essential to restrict initial viral replication and facilitate successful acute clearance [1, 14]. Conversely, persistent exposure to high viral loads during chronic infection induces NK cell exhaustion and dysfunction, characterized by impaired cytotoxicity and cytokine hyporesponsiveness, which correlates with viral persistence and may either alleviate or exacerbate host immunopathology [1, 3].

Unlike T and B cells, which both require antigen presentation for activation [15], NK cell activation is tightly regulated by a balance of germline-encoded activating receptors and inhibitory receptors, enabling discrimination between “self” and “non-self” cells [16]. Through this mechanism, NK cells can be activated when they meet virus-infected cells with downregulated MHC-I molecules or upregulated stress-induced ligands [1, 17]. This innate capacity is particularly critical in the early stages of infection as well as in controlling persistent viral infections by limiting viral spread and modulating immune homeostasis [1]. However, viruses have evolved sophisticated strategies to evade NK cell surveillance [18]. In this review, we systematically elucidate the biological characteristics of NK cells, their multifaceted roles in combating acute and chronic viral infections, and the mechanisms by which viruses evade NK cell control. We also discuss cutting-edge immunotherapeutic strategies against virus infection by modulating NK cell functions. This review aims to provide a cohesive framework for understanding NK cell function in antiviral immunity and identify future directions for translating basic research into clinical applications.

## Biological foundations of NK cell-mediated antiviral immunity

### NK cell development and receptor biology

**Figure 2:**
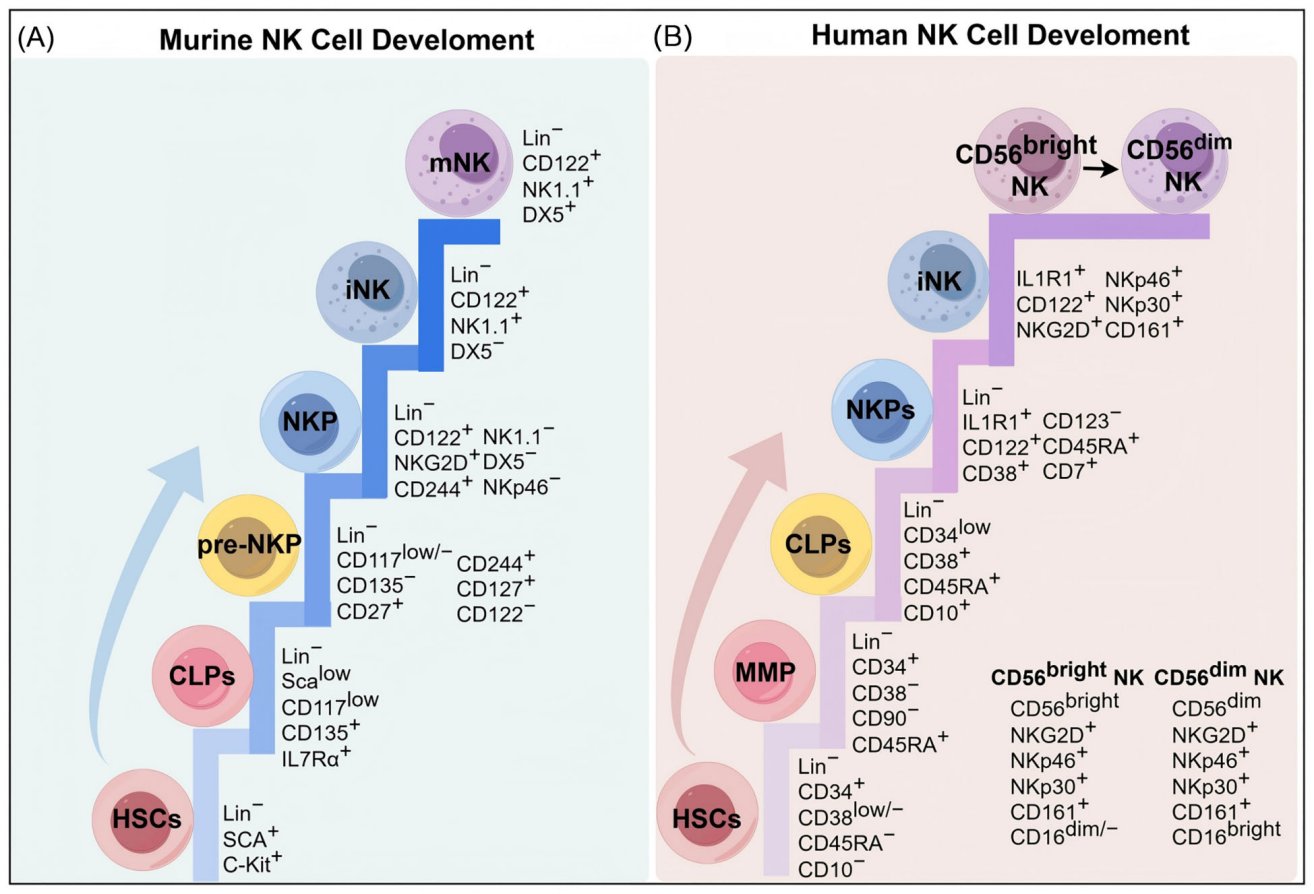
Schematic representation of murine and human NK cell development pathways. This diagram outlines the developmental progression of NK cells in mice and humans, originating from HSCs in the bone marrow. (A) Murine NK cell development proceeds through sequential differentiation stages from CLPs, pre-NKPs, NKPs, and iNK to mNK. Each stage is defined by the progressive acquisition or loss of specific lineage and surface markers. (B) Human NK cell development follows a developmental continuum from HSCs, progressing through MPPs, CLPs, NKPs, and iNKs to immunoregulatory CD56^bright^ NK cells and fully mature cytotoxic CD56^dim^ NK cells. Definitive phenotypic marker combinations characterizing each developmental stage are annotated below the corresponding cells.

In both humans and mice, NK cell lineage specification progresses through a highly regulated developmental process [11, 19]. The detailed phenotypic transitions and specific markers defining these stages are comprehensively illustrated in Fig. 2. A defining molecular milestone across species is the expression of CD122 (the IL-2/IL-15 receptor βchain), which renders developing cells responsive to IL-15, the principal cytokine governing NK cell specification and survival [11]. Upon maturation, human peripheral NK cells are broadly classified into the immunoregulatory CD56^bright^ subset (characterized by robust cytokine production) and the cytotoxic CD56^dim^ subset (characterized by high expression of CD16/FcγRIIIa and potent degranulation capacity) [11]. Similarly, although lacking CD56, mature murine NK cells are functionally delineated by the sequential surface expression of CD27 and CD11b. They transition from an immature immunoregulatory CD11b^−^CD27^+^ stage to a highly cytotoxic, terminally differentiated CD11b^+^CD27^−^phenotype [11].

**Figure 3:**
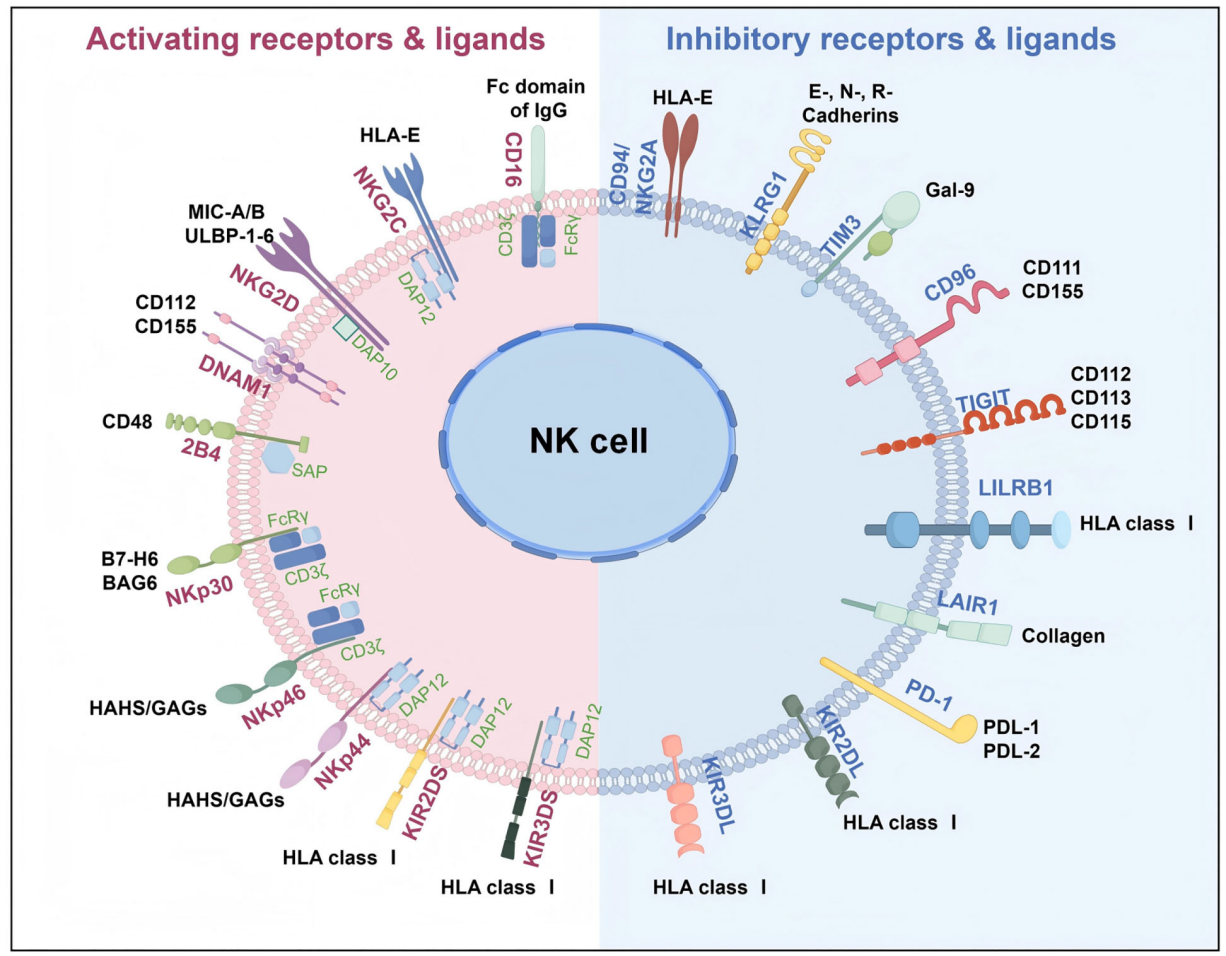
The repertoire of NK cell surface receptors and their cognate ligands. NK cell activation and effector functions are precisely orchestrated by a dynamic integration of signals derived from a diverse array of germline-encoded activating and inhibitory receptors. (Left) Activating receptors, including NCRs (NKp30, NKp44, and NKp46), NKG2D, DNAM-1, and the low-affinity Fc receptor CD16, recognize “altered-self” or “non-self” stress-induced ligands (e.g. MICA/B, ULBPs, and specific viral antigens) upregulated on virus-infected or malignant cells. Lacking intrinsic kinase activity, these receptors predominantly associate with transmembrane adaptor proteins containing ITAMs (e.g. DAP12, FcRγ, and CD3ζ) or YXXM motifs (e.g. DAP10) to transduce downstream cytotoxic and cytokine-secreting signals. Notably, CD16 uniquely binds the Fc domain of IgG to mediate ADCC. (Right) Inhibitory receptors are essential for maintaining immune homeostasis and self-tolerance. Classic inhibitory receptors, such as inhibitory KIRs and the CD94/NKG2A heterodimer, predominantly engage classical and non-classical MHC-I molecules (e.g. HLA class I, HLA-E) constitutively expressed on healthy host cells. Furthermore, a specialized set of immune checkpoint receptors (e.g. TIGIT, TIM-3, PD-1, LAIR1, and LILRB1) bind to their respective broadly expressed ligands (e.g. CD112/CD155, Gal-9, and PD-L1/2) to fine-tune or dampen NK cell hyperactivation. These inhibitory molecules generally suppress NK cell function via ITIMs or ITT-like motifs, which recruit phosphatases to abrogate activation signaling cascades.

Functionally, NK cell activation is governed by a delicate balance between germline-encoded activating and inhibitory receptors, which have been detailly demonstrated in Fig. 3. Briefly, major inhibitory receptors, including killer immunoglobulin-like receptors (KIRs) and the CD94/NKG2A heterodimer, recognize classical and non-classical MHC-I molecules (Table 1), transducing suppressive signals via cytoplasmic immunoreceptor tyrosine-based inhibitory motif (ITIM) to maintain self-tolerance [20]. Conversely, activating receptors, such as natural cytotoxicity receptors (NCRs) and NKG2D, recognize viral glycoproteins and stress-induced ligands, signaling through immunoreceptor tyrosine-based activation motif (ITAM)-containing adapters such as DAP12 or CD3ζ [20]. Notably, MHC-I polypeptide-related sequence A (MICA) and MHC-I polypeptide-related sequence B (MICB) function as MHC-I-related, stress-induced ligands specifically recognized by the activating receptor NKG2D [21]. Structurally and functionally, these MIC proteins are distinct from both classical (HLA-A, -B, -C) and non-classical (HLA-E, -F, -G) human leukocyte antigen (HLA) molecules; MICA/B do not associate with β2-microglobulin (β2m) or present antigenic peptides to T cells [22].

**Table 1:** Key features of MHC-I molecules.

Features	Details
Expression	Nearly all nucleated cells (constitutive)
Primary function	Present endogenous antigens to CTLs
Structure	Heterodimer: transmembrane α chain + β₂-microglobulin
Peptide groove	Formed by α1/α2 domains
Peptide length	8–10 aa
Antigen source	Endogenous (intracellular/viral proteins)
Core pathway	Proteasome degradation → TAP transport (ER) → Assembly → Golgi trafficking
Human homologs	Classical HLA: HLA-A, -B, -C; Non-classical HLA: HLA-E, -F, -G; MHC-I-related (non-HLA): MICA, MICB

Concluding the regulatory landscape of NK cell surface molecules, emerging non-HLA-specific immune checkpoints, namely T cell immunoreceptor with Ig and ITIM domains (TIGIT), T cell immunoglobulin and mucin domain-containing protein 3 (TIM-3), programmed cell death protein 1 (PD-1), leukocyte-associated immunoglobulin-like receptor 1 (LAIR1), and leukocyte immunoglobulin-like receptor B1 (LILRB1), have been recognized as critical determinants of NK cell homeostasis and dysfunction during viral infections (Fig. 3). Rather than operating through classic missing-self recognition, these receptors actively modulate antiviral immunity by engaging specific stress ligands or viral decoys. For instance, the persistent upregulation of TIGIT, TIM-3, and PD-1 during chronic infections [e.g. human immunodeficiency virus type 1 (HIV-1), hepatitis B virus (HBV), and hepatitis C virus (HCV)] potently drives NK cell functional exhaustion and directly predicts poor clinical outcomes, including progression to severe liver fibrosis or hepatocellular carcinoma (HCC) [23–25]. Conversely, LAIR1 acts as a crucial rheostat during acute respiratory infections [such as severe coronavirus disease 2019 (COVID-19)], where its dysfunction exacerbates hyper-inflammation and lung damage [26, 27]. Furthermore, viruses can directly exploit these pathways, as seen with the human cytomegalovirus (HCMV) decoy gpUL18, which specifically engages LILRB1 to facilitate immune evasion [28, 29]. The expression dynamics and genetic variations of these non-classical inhibitory receptors serve as decisive molecular brakes that dictate the clinical trajectory of diverse viral diseases.

### NK cell recognition and antiviral effector functions

**Figure 4:**
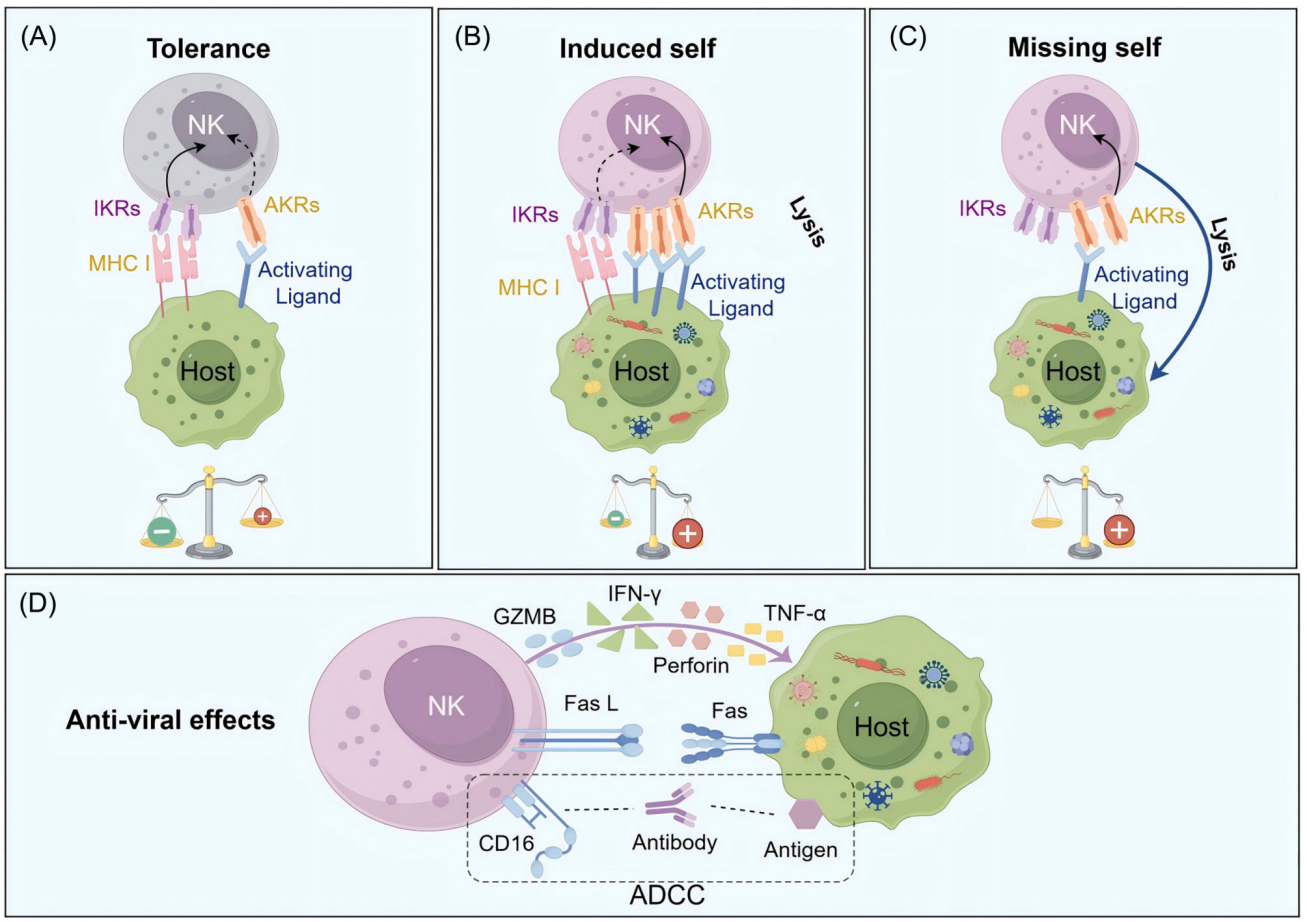
Mechanisms of NK cell recognition and antiviral functions. (A) Immune tolerance: in healthy host tissues, the constitutive expression of MHC-I molecules ensures robust engagement of inhibitory killer receptors (e.g. KIRs, NKG2A), delivering dominant suppressive signals that maintain NK cell quiescence and prevent collateral damage to self-tissues. (B) Induced-self recognition: upon viral infection, cellular stress pathways trigger the aberrant upregulation of stress-induced ligands (e.g. MICA/B, ULBPs) on the host cell surface. These ligands engage activating killer receptors (e.g. NKG2D), actively stimulating NK cell-mediated lysis. (C) Missing-self recognition: some viruses employ evasion strategies to downregulate or ablate surface MHC-I expression to escape T cell detection. This loss of self-ligands abrogates inhibitory signaling, effectively lowering the activation threshold and rendering the infected cell susceptible to NK cell attack. The synergy between “induced-self” and “missing-self” signals shifts the signaling equilibrium toward a pro-cytolytic state. (D) Antiviral effector mechanisms: activated NK cells execute viral clearance through conserved pathways: (i) cytokines generation: the secretion of effector cytokines (e.g. IFN-γ, TNF-α) from activated NK cells could inhibit viral replication or restricts viral dissemination by modulating the local immune microenvironment and priming adaptive immune responses; (ii) granule exocytosis: the directed release of perforin and granzymes (GZMB) into the immunological synapse to induce osmotic lysis and caspase-dependent apoptosis; (iii) death receptor signaling: the engagement of Fas (CD95) or TNF receptors on target cells by NK cell-expressed ligands (FasL, TRAIL, or TNF-α), initiating apoptotic cascades; and (iv) ADCC: CD16-mediated recognition of the Fc fragment of antibodies bound to viral antigens, triggering potent NK cells cytotoxicity.

Unlike T and B lymphocytes, NK cell activation does not require prior antigen sensitization; instead, it relies on a dynamic balance of germline-encoded receptors [16]. Under physiological conditions, the constitutive engagement of self MHC-I molecules, the structural and functional features of which are summarized in Table 1, delivers dominant inhibitory signals to maintain NK cell quiescence [30]. During viral infections, NK cells break this tolerance through two complementary recognition pathways, as illustrated in Fig. 4. First, in the “missing-self” recognition model, viruses often downregulate host MHC-I expression to evade CD8^+^ T-cell clearance (e.g. via herpesvirus proteins like HCMV US2 or US11) [31]. This loss of self-ligands removes the inhibitory “brakes” on NK cells, significantly lowering their activation threshold [6]. Second, the cellular stress induced by viral replication triggers “induced-self” recognition, characterized by the aberrant upregulation of stress ligands (e.g. MICA/B) that directly engage activating receptors such as NKG2D [21]. The synergy between these pathways profoundly shifts the immunological balance toward a pro-cytolytic state [32].

Upon target recognition, NK cells execute viral clearance through coordinated cytolytic and immunomodulatory mechanisms (Fig. 4). Direct cytotoxicity is achieved via the targeted exocytosis of perforin- and granzyme-containing granules to induce target cell apoptosis [33], alongside FasL or TRAIL [34]. Beyond direct lysis, activated NK cells critically shape the broader antiviral immune landscape by rapidly secreting pro-inflammatory cytokines (predominantly IFN-γ and TNF-α) and chemokines (e.g. CCL3, CCL4, and XCL1) [35]. Specifically, NK cell-derived IFN-γ impedes viral replication and upregulates MHC-I expression on bystander cells [36], while chemokine gradients facilitate the recruitment of conventional type 1 dendritic cells (cDC1s) [37]. This pivotal NK-DC crosstalk promotes DC maturation and the subsequent priming of downstream CD8^+^ T-cell responses, effectively bridging innate and adaptive antiviral immunity [11].

### Adaptive features and memory-like responses of NK cells

Although traditionally categorized as innate immune effectors, NK cells have been recognized to exhibit adaptive immune characteristics during viral infections [8, 38]. This paradigm shift highlights that specific NK cell subsets can undergo antigen-driven clonal-like expansion, develop long-lived immunological memory, and mount enhanced recall responses upon secondary challenge [38–40]. Currently, adaptive NK cell responses are predominantly categorized into two forms. The first is pathogen-induced antigen-specific memory [41], which is usually triggered by specific viral antigens, including UL40 peptides in HCMV infection [42], Env and Gag epitopes in HIV-1 infection [43], Nucleoprotein (NP) motifs in influenza A virus (IAV) infection [43], HBsAg/HBcAg in HBV infection [44], and non-structural protein 1 (NS1) NS1 proteins in ZIKV infection [45]. The detailed phenotypic remodeling and antiviral mechanisms of these virus-specific memory NK cells will be discussed in their respective viral sections below. The second form is cytokine-induced memory-like (CIML) NK cells [46]. Triggered by short-term pre-activation with pro-inflammatory cytokines (primarily IL-12, IL-15, and IL-18), CIML NK cells undergo profound epigenetic reprogramming (such as demethylation of the IFN-γ gene enhancer) and upregulate the high-affinity IL-2 receptor CD25, which allows them to escape KIR-mediated inhibition and acquire long-lasting memory-like properties [47–49]. Even after a prolonged resting phase, CIML NK cells exhibit significantly enhanced IFN-γ secretion, robust cytotoxicity, and prolonged *in vivo* persistence against viral infections and malignancies [47, 50]. These attributes have successfully translated to the clinic, where adoptive transfer of donor-derived CIML NK cells demonstrated robust expansion, sustained persistence, and therapeutic efficacy in patients with myeloid leukemia relapse after allogeneic transplantation without inducing severe graft-versus-host disease (GVHD) [51], establishing them as a validated platform for next-generation immunotherapy.

## Dynamic responses of NK cells to viral infections

**Figure 5:**
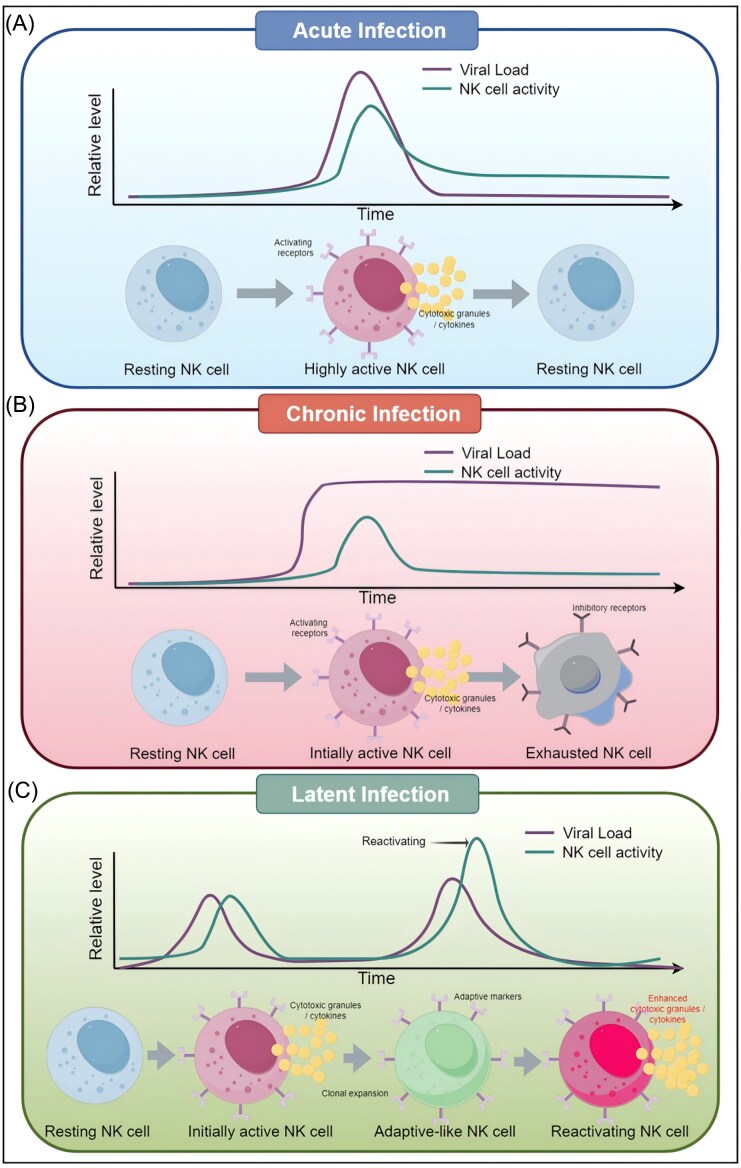
Dynamic alterations of NK cell responses across distinct viral infections. NK cell activation, effector functions, and phenotypic evolution are strictly shaped by the specific infection pattern. (A) Acute infection: Resting NK cells are rapidly activated to restrict viral replication via the release of cytotoxic granules and cytokines, returning to a resting state upon viral clearance. (B) Chronic infection: Despite initial activation, sustained high viral loads and continuous antigenic stimulation drive NK cells into functional exhaustion, characterized by diminished effector capacities and aberrant upregulation of inhibitory receptors. (C) Latent infection: Periodic viral reactivations drive profound functional remodeling of NK cells. This recurrent antigenic exposure triggers clonal expansion to generate long-lived “adaptive-like” NK cells, which mount a superior, virus-specific recall response with enhanced cytotoxicity upon subsequent reactivations.

Upon encountering virus-infected cells, NK cells integrate signals from germline-encoded inhibitory and activating receptors to execute precise elimination via “missing-self” and “induced-self” recognition [6, 30, 52]. This recognition efficiently triggers the anti-viral responses of NK cells as illustrated above, including the conserved cytotoxic effector pathways and immune regulation functions [17]. However, rather than mounting a uniform response, the kinetics, effector magnitude, and phenotypic remodeling of NK cells are highly dynamic and strictly dictated by the specific virus species (Fig. 5) [1]. Notably, while we categorize these host-pathogen interactions into acute, chronic/persistent, and latent/reactivating infections to establish a practical organizational framework, this division is not an absolute biological classification. In clinical reality, these pathogenic states often overlap and transition fluidly. For instance, HBV can manifest as either an acute self-limiting infection or progress into a lifelong chronic phase [53], whereas herpesviruses such as HCMV and Epstein–Barr virus (EBV) navigate continuously between acute primary infection, asymptomatic latency, and periodic clinical reactivation phases [1, 54].

In acute viral infections (see Section “Virus induced acute infections” below), NK cells mainly undergo rapid, cytokine-driven activation to reach peak functional activity, promptly restricting viral dissemination and shaping early adaptive immunity [1]. In contrast, during chronic and persistent infections (see Section “Virus induced chronic or persistent infections” below), sustained high viral loads and localized immunosuppressive microenvironments subject NK cells to continuous antigenic stimulation, which drives initially active NK cells into functional exhaustion, a state characterized by diminished cytolytic capacity, skewed subset distributions, and the aberrant upregulation of inhibitory checkpoints [1]. In latent and reactivating infections (see Section “Virus induced latent/reactivating infections” below), host-virus co-evolution imposes a unique selection pressure. Periodic viral reactivations drive profound epigenetic and transcriptional rewiring in NK cells, culminating in the generation of antigen-driven, long-lived “adaptive” or “memory-like” subsets endowed with superior, virus-specific recall responses [1, 38].

Importantly, NK cell activation is not always beneficial, acting as a double-edged sword that depends heavily on the infection stage, the tissue microenvironment, and the host’s inflammatory state [1, 55]. While early NK cell activation is protective and limits initial viral replication, dysregulated or prolonged activation in later stages can become harmful [55]. For example, in severe COVID-19, hyper-activated NK cells in the lungs release excessive pro-inflammatory cytokines, which worsens lung injury and drives complications such as acute respiratory distress syndrome (ARDS) [56]. Similarly, during acute HBV flares, over-activated CD56^bright^ NK cells in the liver express high levels of death ligands, such as TRAIL and FasL [57]. Instead of clearing the virus, these cells trigger the apoptosis of healthy, neighboring liver cells, directly causing severe liver damage [57, 58]. Thus, the therapeutic outcome of activating NK cells depends entirely on the clinical context, and treatments must be carefully balanced to prevent tissue damage.

**Figure 6:**
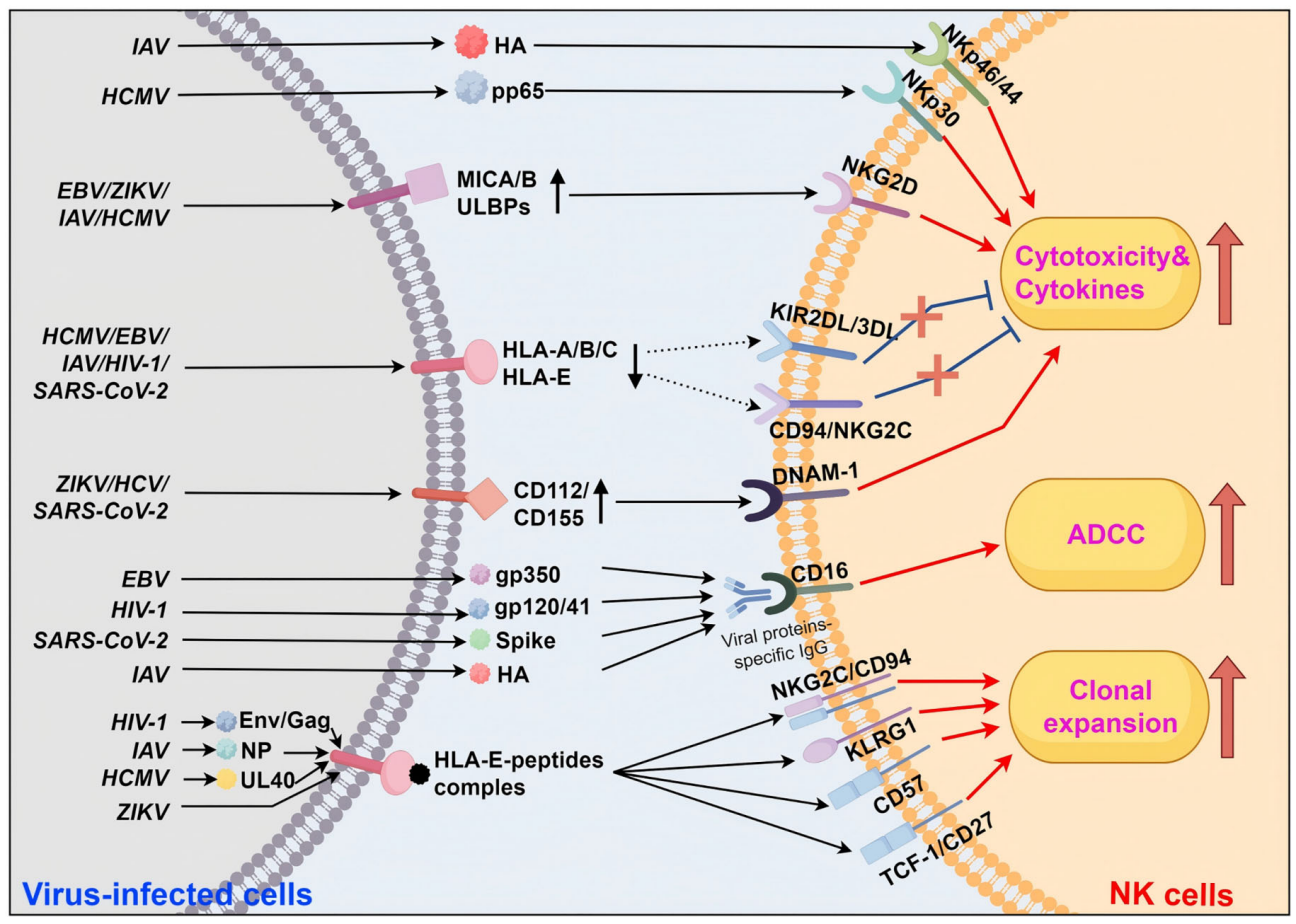
The mechanisms of specific viral infections driving NK cell responses. NK cells execute antiviral immunity through distinct functional pathways based on specific receptor-ligand interactions during different viral infections. Direct viral glycoproteins (e.g. IAV HA) and “induced-self” stress ligands (e.g. MICA/B, ULBPs) engage activating receptors (NKp46, NKp44, NKp30, or NKG2D) to trigger cytotoxicity and cytokine secretion. Simultaneously, the downregulation of MHC-I molecules (“missing-self”) (e.g. HLA-A/B/C/E) releases KIR-mediated inhibition. Besides, the low-affinity Fc receptor CD16 recognizes virus-specific IgGs bound to viral surface antigens (e.g. EBV gp350, HIV-1 gp120/41, SARS-CoV-2 Spike, and IAV HA), mediating robust ADCC. Moreover, specific viral peptides (e.g. HCMV UL40, IAV NP, and HIV-1 Env/Gag) presented by HLA-E, along with distinct viral encounters (e.g. ZIKV), drives antigen-specific clonal expansion and the generation of memory-like NK cells characterized by specific phenotypic markers (such as KLRG1, CD57, or TCF-1/CD27).

In the following sections, we systematically dissect these distinct, virus-specific mechanisms to decipher how diverse virus shape NK cell immunity and highlight their corresponding immunotherapeutic targets (Fig. 6).

### Virus induced acute infections

Acute viral infections are typically characterized by rapid viral replication, broad tissue tropism, and the induction of robust, systemic inflammatory responses. In the critical temporal window before adaptive immunity is fully primed, NK cells serve as the indispensable first line of defense, rapidly mobilizing to primary sites of infection to restrict viral dissemination [1, 59]. In this context, NK cells exert dual functionalities: they directly execute rapid cytotoxicity against infected cells and orchestrate the early inflammatory milieu or tissue repair processes through the coordinated secretion of cytokines, chemokines, as well as via direct cell-to-cell crosstalk [1, 59]. The subsequent sections dissect these dynamic, rapid-onset responses across representative acute viral models.

#### Influenza virus

Influenza virus represents a prototypical acute respiratory infection characterized by rapid viral replication in the respiratory tract [60]. It is a highly mutable single-stranded RNA virus of the Orthomyxoviridae family, causing annual global epidemics with ~1 billion infections and 500 000 deaths annually [61]. Among the three major subtypes (A, B, and C), IAV is the most clinically relevant due to its frequent antigenic drift/shift and ability to trigger severe pandemics, making it a top priority for clinical management and basic research [60]. NK cells serve as an indispensable first line of defense, rapidly migrating to the infected lungs to execute a delicate balance between robust viral clearance and the prevention of excessive immunopathology [62]. Clinically, the quantitative and functional dynamics of NK cells strongly correlate with IAV severity and patient prognosis [63, 64]. Acute IAV infection frequently induces a profound decline in circulating NK cells (NK lymphopenia), which is particularly pronounced in critically ill cases due to active pulmonary recruitment and virus-induced cell death [63, 65]. Further analyses reveal that impaired NK cell cytotoxicity, characterized by diminished IFN-γ, perforin, and granzyme B release, serves as a key predictor of respiratory failure and delayed viral clearance [64].

Central to the direct cytolytic response is the NCR NKp46 (murine ortholog Ncr1), which uniquely and conservatively interacts with the viral hemagglutinin (HA) glycoprotein [66]. This recognition is predominantly mediated by α2,6-terminal sialic acid (SA) residues modified on the Thr225 residue of the membrane-proximal immunoglobulin-like domain of NKp46, which is the critical site governing HA binding and receptor activation [67, 68]. Following this specific HA-NKp46 interaction, the receptor transduces intracellular activating signals via its non-covalently associated ITAM-bearing adapters, such as CD3ζ and FcεRIγ, unleashing robust degranulation and IFN-γ production to lyse infected epithelial cells [69, 70]. The non-redundant nature of this pathway is underscored by the uniform lethality observed in Ncr1-deficient mice following IAV challenge [68].

Lung-resident NK cells orchestrate a highly specialized defense program. The unique lung-resident CD56^bright^CD49a^+^CD69^+^CD103^+^ NK cell subset mounts robust IAV-specific cytotoxic responses in human lung explants [71]. Beyond direct cytotoxicity, conventional (RORγt-negative) NK cells in the respiratory tract exert a vital tissue-protective role during IAV infection by secreting the regenerative cytokine IL-22 [72, 73]. By engaging constitutively expressed IL-22 receptors on airway epithelial cells [74], NK-derived IL-22 drives the proliferation and repair of damaged epithelial monolayers, thereby restoring mucosal barrier integrity and mitigating excessive pulmonary inflammation and injury [72, 73]. The non-redundant protective role of NK-derived IL-22 has been elegantly confirmed *in vivo*. Specifically, transferring IL-22-sufficient WT NK cells into IAV-infected IL-22-deficient (*il-22^−^*^/−^) mice successfully rescued the impaired epithelial regeneration and mitigated the severe lung inflammation [72]. Crucially, in parallel control experiments, the transfer of IL-22-deficient NK cells (or cyclophilin D-deficient NK cells with impaired IL-22 production) into *il-22^−^*^/−^ mice failed to provide such restoration [73], confirming that the protective effect is strictly dependent on NK cell-derived IL-22.

In addition to these innate responses, recent evidence reveals that NK cells can develop antigen-specific memory against IAV [43]. This adaptive-like recall response is strictly orchestrated via the CD94/NKG2C receptor, which specifically recognizes HLA-E-binding nonameric peptides derived from the IAV nucleoprotein (NP) (e.g. TMDSNTLEL) [43]. Upon re-exposure to these viral antigens, the memory NK cell subset, characteristically marked by a KLRG1^hi^α4β7^hi^NKG2C^+^ phenotype, mounts a potent, epitope-specific secondary responses, but they can’t cross-react to mismatched peptides [43]. Furthermore, HA-NKp46 interactions also drive antigen-specific memory formation. In murine models, intranasal IAV infection induces a memory NK cell subset expressing NKp46 and NKG2A that specifically recognizes N-linked glycosylation sites on the viral HA protein, driving pulmonary recruitment and viral control upon re-challenge [75]. These antigen-specific memory NK cells exhibit remarkable longevity, stably persisting in the human peripheral blood for years after the initial influenza exposure, to provide long-lasting immunosurveillance [43, 45, 75].

#### Severe acute respiratory syndrome coronavirus 2

Severe acute respiratory syndrome coronavirus 2 (SARS-CoV-2) is a highly transmissible coronavirus with an estimated basic reproduction number (*R*₀) of 2.5–3.5 in its early phase and substantial genetic variability [76]. The global average case fatality rate approximates 1.2%, and during the peak of the pandemic in 2021, COVID-19 emerged as a leading cause of death worldwide [77, 78]. As a single-stranded RNA virus responsible for COVID-19, SARS-CoV-2 is predominantly controlled by the innate immune system [79, 80], in which NK cells occupy a central role through mechanisms, including direct cytotoxicity, immunomodulation, and cellular crosstalk to limit viral replication and shape clinical outcomes [81]. Peripheral NK cell lymphopenia is a hallmark of severe COVID-19, with a sustained reduction in total NK cell count observed in critically ill patients [82]. Peripheral NK cells from patients with severe COVID-19 produced low levels of IFN-γ and TNF related to those from ambulant patients with COVID-19. Single cell RNA-sequencing data revealed that NK cells from patients with severe COVID-19 showed reduced expressions of genes associated with effector molecules, which is correlated with high levels of TGF-β related signaling [56]. Several studies suggest that the reduced peripheral NK cells may partially be attributed to the recruitment of circulating NK cells to infected lung tissues driven by chemokine gradients, including CCL2, CXCL9, and CXCL10 [82]. However, these recruited NK cells may be harmful to lung tissues, because studies reveal that they secrete excessive pro-inflammatory cytokines that exacerbate respiratory tissue injury and drive severe immunopathology, such as ARDS, rather than executing viral clearance [83, 84]. Besides, the systemic reduction is accompanied by a profound remodeling of the remaining peripheral NK cell pool in the blood. Specifically, the immunoregulatory CD56^bright^ subset undergoes a more drastic depletion compared to the CD56^dim^ subset, resulting in a markedly elevated CD56^dim/^CD56^bright^ ratio, which is positively correlated with disease severity, serving as a robust prognostic biomarker strongly linked to adverse clinical outcomes [85]. The impact of this remodeling is further manifested in functional failure, as the remaining CD56^dim^CD16^+^ NK subset exhibits severely impaired lytic capacity and perforin/granzyme secretion in severe cases [86], while the contracted CD56^bright^CD16^−/dim^ subset shows a significant loss of its immunomodulatory cytokine-producing capacity, leading to a profound functional imbalance between cytotoxic and immunomodulatory NK cell compartments [86]. This disrupts critical NK-DC crosstalk, thereby indirectly impairing DC maturation and the subsequent priming of downstream adaptive T-cell responses. Consequently, the failure to establish coordinated cytotoxic T-lymphocyte (CTL) and antibody-mediated viral clearance allows persistent SARS-CoV-2 replication, which drives a feedback loop of continuous inflammatory myeloid cell recruitment and ultimately fuels the severe systemic cytokine storm [86–88]. Dysregulated expression of immune checkpoint receptors drives NK cell functional exhaustion during severe SARS-CoV-2 infection: inhibitory receptors, including NKG2A, KIR2DL1, PD-1, and TIM-3 are significantly upregulated, whereas activating receptors such as NKG2D, NKp30, NKp46, and DNAM-1 are consistently downregulated, which collectively shifts the signaling balance toward immune suppression and blunts NK cell antiviral reactivity [89–91].

Interestingly, studies have revealed that the non-structural protein 13 (Nsp13) of SARS-CoV-2 contains a 9-mer peptide (VMPLSAPTL) that stabilizes HLA-E expression but, unlike host self-peptides, fails to trigger the inhibitory signaling of CD94/NKG2A [92]. This unique “molecular mismatch” effectively creates a window for NK cell activation, theoretically allowing NKG2A^+^ NK cells to bypass viral suppression and clear infected lung epithelial cells [92]. Yet, in clinical practice, this protective mechanism appears to be insufficient in severe cases. Longitudinal cohorts have demonstrated that the persistent high expression of NKG2A on NK cells is strongly associated with higher viral loads, severe lung injury, and poor patient prognosis [83, 92, 93]. This suggests that while the Nsp13-HLA-E axis provides a potential avenue for immune attack, the overwhelming systemic exhaustion and the redundant inhibitory signals in the severe COVID-19 microenvironment eventually neutralize this advantage, leading to immune failure.

#### Zika virus

ZIKV, a mosquito-borne orthoflavivirus, poses significant global health risks due to its neurotropism and capacity to cause congenital anomalies [94, 95]. During acute ZIKV infection, circulating NK cells undergo robust activation and phenotypic remodeling. Mass cytometry (CyTOF) profiling of patient cohorts reveals that acute-phase NK cells shift toward a terminally differentiated CD57^+^NKG2C^+^KIR3DL1^+^ phenotype, with a functional bias toward cytokine production (primarily IFN-γ and TNF-α) over direct cytotoxicity [96]. Beyond circulation, tissue-resident NK cells establish crucial protective barriers. At the maternal-fetal interface, decidual NK (dNK) cells eliminate ZIKV-infected trophoblasts; accordingly, *in vivo* NK-cell depletion significantly elevates placental viral loads and exacerbates fetal intrauterine growth restriction (IUGR) [97]. Similarly, during neurotropic infection, NK cells rely on the checkpoint receptor LILRB4 (gp49B) to resolve neuroinflammation, the deficiency of which impairs viral clearance in the brain, accelerating neurodegeneration and resulting in over 70% mortality [98].

Beyond these acute response dynamics, NK cells not only execute classical innate defense mechanisms but also develop a unique subset of antigen-specific memory-like NK cells with stem cell-like properties [99]. These specialized cells, termed NK memory stem cells (NKSCMs), are characterized by a TCF-1^hi^CD27^+^ phenotype [99]. Upon secondary ZIKV challenge, NKSCMs undergo robust, virus-specific clonal expansion and differentiate into highly functional effector NK cells that exert superior antiviral protection compared to naive NK subsets [99]. The generation, self-renewal, and maintenance of these adaptive features are driven by the Wnt/β-catenin-TCF-1 signaling pathway, which orchestrates the transcriptional programs governing both immunological memory and stemness in NK cells [99]. However, several key questions remain regarding the nature of this memory. Unlike the peptide-specific NK memory identified in HCMV or HIV models, the specific ZIKV antigens responsible for driving NKSCM formation have not yet been identified. Furthermore, while the recall response provides enhanced protection against ZIKV, it remains to be determined whether this memory is strictly antigen-specific or represents a broader form of CIML, as evidence from heterologous viral challenges is currently lacking in the ZIKV context [99].

### Virus induced chronic or persistent infections

The transition from an acute viral encounter to a chronic infection is inherently coupled with the gradual failure of host immune clearance mechanisms, allowing pathogens to establish sustained replication and persistent antigenic stimulation [100]. In the setting of chronic viral infections, the constant exposure to high viral loads, alongside the establishment of a localized immunosuppressive microenvironment, exacts a profound toll on NK cell functions [101]. This is defined by progressive NK cell exhaustion, characterized by skewed subset redistributions, downregulated activating cascades, and the aberrant overexpression of inhibitory immune checkpoints [57, 101, 102]. Consequently, NK cells are stripped of their intrinsic cytolytic and cytokine-producing capacities, shifting from antiviral guardians to tolerant bystanders [57, 101, 102].

A critical evaluation of persistent viral settings reveals that NK cell exhaustion is driven by a synergistic combination of intrinsic metabolic paralysis and extrinsic microenvironmental suppression. Intrinsically, chronic antigen exposure triggers TOX-dependent transcriptional rewiring, leading to severe mitochondrial defects, disrupted oxidative phosphorylation (OXPHOS), and impaired glycolysis, which ultimately compromise effector cytokine synthesis and degranulation [103–105]. Extrinsically, this cellular exhaustion is actively reinforced by the immunosuppressive tissue microenvironment. Recruited suppressor cells, such as regulatory T cells (Tregs) and Myeloid-derived suppressor cells (MDSCs), secrete TGF-β and IL-10 to directly downregulate NK-activating receptors, while simultaneously depleting extracellular nutrients and producing reactive oxygen species via arginase-1 expression [1, 3, 106]. Consequently, resolving chronic viral infections demands therapeutic strategies capable of simultaneously reversing intrinsic metabolic exhaustion and bypassing extrinsic microenvironmental barriers.

#### Hepatitis B virus

HBV, a partially double-stranded relaxed circular DNA virus of the Hepadnaviridae family, poses a major global health challenge, affecting ~240 million individuals worldwide and remaining a critical public health concern [107]. Accumulating *in vivo* evidence has defined the non-redundant role of NK cells in the host immune response against HBV infection [108]. In a well-established hydrodynamic transfection mouse model of HBV replication, genetic ablation of NK cells resulted in the persistent presence of the HBV transcriptional template in the liver for at least 60 days, directly demonstrating that NK cells are indispensable for the complete elimination of intrahepatic HBV transcriptional templates [109]. During the clinical course of HBV infection, NK cells undergo dynamic quantitative and qualitative shifts that dictate the balance between viral clearance and hepatocyte damage. In acute, self-limiting HBV infection, peripheral NK cell frequencies rise transiently prior to the peak of alanine aminotransferase (ALT), facilitating rapid viral containment [57]. Conversely, chronic HBV (CHB) infection is characterized by a significant reduction in the absolute numbers of both circulating and intrahepatic NK cells [58, 108].

Evidence has demonstrated that the functional activity of the NKG2C^+^ NK cell subset is tightly correlated with effective control of HBV replication [110]. These potent NKG2C^+^ NK cells improve HBV control by exhibiting superior effector functions compared to the inhibitory NKG2A^+^ counterparts [110]. Particularly in individuals with acute HIV–HBV co-infection, improved NKG2C⁺ NK cell function markedly reduces HBV viral load and facilitates the serological clearance of both HBeAg (serves as a potent immunomodulator to induce T-cell tolerance) and HBsAg (facilitates viral entry and acts as an immunological decoy to promote persistence) [110].

Beyond their conventional innate effector functions, HBV exposure can elicit profound adaptive NK cell memory [44, 45]. Recent studies have demonstrated that HBV vaccination induces long-lived memory NK cells specific for the HBsAg [44]. Concurrently, chronic HBV infection generates memory NK cells capable of targeting the HBcAg [44]. These human HBV-specific memory NK cells are phenotypically characterized by a CD56^dim^CD57^+^KLRG1^+^ profile [44]. Upon re-exposure to autologous antigen-presenting cells *in vitro*, these memory NK cells exert superior antiviral effects and robust degranulation, which is primarily driven by NKG2D-dependent cytotoxicity rather than classical innate pathways [44].

As HBV infection progresses to chronicity, persistent high-level viral antigenemia drives profound NK cell exhaustion, thereby facilitating long-term viral persistence [55, 111]. Sustained high-level viral antigenemia is a primary driver of NK cell exhaustion, where circulating HBsAg directly dampens NK cell effector functions and metabolic fitness by competitively binding to the IL-15 receptor β (CD122), which abrogates the IL-15/mTOR signaling axis and impairs essential glucose metabolism [112–114]. In parallel, HBeAg indirectly compromises NK cell antiviral immunity by engaging Toll-like receptor 2 (TLR2) to activate neutrophils; these activated neutrophils upregulate the inhibitory ligand programmed death-ligand 1 (PD-L1), which directly binds to PD-1 on NK cells to deliver suppressive signals, subsequently dampening the capacity of NK cells to produce critical effector cytokines, such as IFN-γ and TNF-α [115]. Concurrently, this functional exhaustion is heavily reinforced by the aberrant upregulation of multiple inhibitory checkpoints, which severely disrupts NK cell signaling homeostasis. For instance, NKG2A is significantly upregulated on NK cells from patients with chronic HBV infection, and blockade of the NKG2A-HLA-E ligand-receptor axis effectively rescues NK cell cytotoxicity and cytokine secretory capacity [116]; the TIGIT signaling pathway also negatively modulates NK cell activation and effector function to promote chronic HBV progression [117]. Furthermore, enhanced expression of immunoglobulin-like transcript 2 (ILT2) on the CD56^dim^CD16^+^ NK cell subset, the predominant cytotoxic population in peripheral circulation, has been identified as an additional critical mediator of NK cell dysfunction, as ILT2 ligation markedly blunts NK cell cytolytic activity against HBV-infected hepatocytes [118].

Compounding the intrinsic functional defect above, the immunosuppressive hepatic microenvironment established during chronic HBV infection further propagates NK cell dysfunction via a network of soluble immunomodulatory factors [57, 116, 119]. Specifically, elevated TGF-β in the HBV-infected liver has been shown to downregulate NK cell activating receptors (e.g. NKG2D and NKp30), while Arginase-1, predominantly produced by MDSCs, impairs NK cell metabolic fitness and effector functions [119, 120]. Tregs, a pivotal immunosuppressive population in chronic HBV infection, secrete the anti-inflammatory cytokine IL-10 to specifically drive functional impairment in NKG2A^+^ NK cells, which express significantly higher levels of IL-10R compared to their NKG2A^−^ counterparts, rendering them hypersensitive to IL-10 [116]. In a chronic HBV transgenic mouse model, extracellular Galectin-3, which is highly abundant in the HBV-infected liver, binds to Integrin β1 (ITGB1, also known as CD29) expressed on the surface of conventional hepatic NK cells, driving robust IL-10 production, which not only exacerbates the local immunosuppressive hepatic milieu but also correlates with the progression of HBV-related HCC [121].

Driven by the multi-layered immunosuppressive milieu above, the NK cell compartment undergoes profound structural remodeling in both peripheral blood and hepatic tissue. This dysregulation is manifested by the pathological expansion of the CD56^−^CD16^+^ NK subset, which harbors severe defects in effector functions and represents a key cellular basis for systemic immune impairment [122]. Concurrently, an aberrant accumulation of the immunoregulatory CD5^bright^ NK subset correlates with chronic disease progression and hepatic injury [123, 124]. Collectively, while NK cells exert robust antiviral functions to facilitate viral clearance during acute HBV infection, their progressive subset skewing and functional exhaustion in the chronic phase ultimately transition them into tolerant bystanders, thereby promoting persistent viral existence [125].

#### Hepatitis C virus

HCV, an RNA virus, is a leading cause of chronic viral hepatitis, cirrhosis, and HCC [126, 127]. It continues to pose a major global health burden, with an estimated 56.8 million viraemic infections worldwide; despite 12.7 million patients receiving curative direct-acting antivirals, progress remains insufficient to meet WHO elimination targets by 2030 [128, 129]. Clinical cohorts have demonstrated that patients who successfully resolve acute HCV spontaneously exhibit early, robust activation of NK cells with highly efficient IFN-γ production [130, 131]. Conversely, a weak or delayed NK cell activation, coupled with early upregulation of the inhibitory receptor NKG2A, is associated with chronic viral persistence [130].

Hepatic Kupffer cells act as accessory cells sensing the virus via phagocytic HCV uptake, where viral RNA triggers MAVS-dependent RIG-I signaling to secrete innate cytokines (IFN-β, IL-12, IL-18, and TNF-α), which activate NK cells through paracrine activation of the IFNAR-JAK-STAT1 axis, followed by upregulating the expression of NK cell antiviral effector molecules and cytotoxicity [132, 133]. Moreover, monocytes can sense cells containing HCV replicons, activate the NALP3 inflammasome to cleave pro-IL-18 into mature bioactive IL-18 via caspase-1, and secrete this cytokine to specifically act on CD56^bright^ NK cells to drive robust IFN-γ production and antiviral activity, as the this NK subset predominantly expresses the high-affinity IL-18 receptor (IL-18R) compared to the CD56^dim^ subset [134, 135]. Notably, pre-stimulating NK cells with exogenous IFN-α enables them to kill HCV-infected hepatoma cells through upregulating TRAIL expression, particularly on the CD56^bright^ subset, to induce target cell apoptosis and driving NK cell activation by promoting DNAM-1 (complemented by NKG2D) recognition of CD155/CD112 ligands on hepatoma cells [136, 137].

Chronic HCV infection drives profound alterations in the frequency, subset distribution, and functional integrity of NK cells. The transition from acute to chronic HCV infection is frequently attributed to the virus’s ability to overwhelm early innate responses and establish a state of immune exhaustion characterized by impaired NK-cell signaling [102, 130]. Following the establishment of this chronic phase, the frequency of circulating NK cells in peripheral blood is frequently reduced [130, 138], whereas the number of intrahepatic CD3^−^CD56^+^ NK cells is significantly increased [135]. Within this intrahepatic environment, the CD56^bright^ NK subset undergoes pathological expansion; however, these cells are functionally inhibited, exhibiting severe defects in the production of antiviral cytokines, particularly IFN-γ and TNF-α [138, 139]. Meanwhile, the functional decline extends to the CD56^dim^ subset, where the primary degranulation capacity is also significantly compromised [138, 139]. Furthermore, NK cell-mediated antibody-dependent cellular cytotoxicity (ADCC) is severely impaired, primarily due to the metalloproteinase-mediated shedding of the CD16 receptor [140, 141]. This proteolytic cleavage is a consequence of chronic HCV-induced continuous activation, which upregulates and activates A Disintegrin and Metalloproteinase 17 (ADAM17) on the NK cell membrane. Active ADAM17 cleaves the membrane-proximal stalk region of CD16 (FcγRIIIa), resulting in the shedding of its antibody-binding ectodomain and effectively stripping the functional Fc receptor from the cell surface [140–143]. Besides, NK cells from patients with chronic HCV infection consistently show upregulated expression of inhibitory receptors (e.g. NKG2A), accompanied by downregulation of key activating receptors including NKG2D, NKp30, and NKp46 [138, 139]. This dysregulated receptor repertoire disrupts the balance between activating and inhibitory signaling cascades, thereby directly suppressing NK cell effector cytotoxic function against HCV.

#### Human papillomavirus

Human papillomavirus (HPV), a double-stranded circular DNA virus with tropism for skin and mucosal epithelial cells, induces a spectrum of diseases ranging from benign proliferative lesions (e.g. warts) to malignant tumors [144–146]. High-risk human papillomavirus (Hr-HPV) subtypes, predominantly HPV16 and HPV18, are responsible for ~70% of global cervical cancer cases [144, 147]. Beyond gynecological malignancies, these oncogenic genotypes are also etiologically linked to a rapidly rising subset of head and neck squamous cell carcinomas, predominantly oropharyngeal squamous cell carcinoma [148, 149]. HPV drive oncogenesis mainly through the expression of E6 and E7 oncoproteins, which not only disrupt the p53 and retinoblastoma (Rb) tumor suppressor pathways to induce uncontrolled cell proliferation and genomic instability, but also establish a multifaceted immune evasion program to escape host immune clearance [149]. As Hr-HPV infection progresses from persistent cervical dysplasia to invasive malignancy, both the quantitative abundance and functional integrity of NK cells undergo a systemic and progressive decline [150]. Multi-center clinical cohorts have demonstrated a stepwise reduction in the frequencies of circulating CD56^+^ NK cells across chronic cervicitis, low-to-high-grade cervical intraepithelial neoplasia (CIN I–III), and invasive cervical cancer, where the lowest peripheral NK cell counts strongly correlate with advanced clinical FIGO stages and lymph node metastasis [151, 152].

In the majority of individuals, HPV infection is transient and spontaneously cleared within 12–24 months, a process where NK cells serve as the frontline defense [153, 154]. In regressing lesions, high levels of NK cell infiltration and elevated expression of activating receptors like NKp46 and NKG2D are positively correlated with successful viral eradication [149, 155]. Furthermore, NK-derived IFN-γ and TNF-α suppress the transcription of viral E6/E7 mRNA as well as orchestrate a robust Th1-polarized response, which both favor self-healing [149, 156]. However, the immunological outcome, spontaneous clearance versus viral persistence, is primarily governed by the individual’s genetic susceptibility and the functional state of the local immune microenvironment [154, 157]. Genetically, individuals who spontaneously clear HPV infections often harbor favorable immunogenetic profiles, such as the co-existence of inhibitory KIR receptors (e.g. KIR3DL1 or KIR2DL1) and their cognate HLA class I ligands [52, 158]. This genetic combination ensures the presence of a high frequency of functionally educated (“licensed”) NK cells, which mount vigorous cytolytic and polyfunctional cytokine responses upon viral challenge to prevent lesions from progressing to invasive cancer [52, 158]. Conversely, a transition toward chronic or latent infection is more frequent in individuals who exhibit an “exhausted” NK cell profile, characterized by the aberrant upregulation of inhibitory receptors such as NKG2A, TIGIT, and TIM-3 [149, 159]. In these persistent cases, the local milieu is often dominated by immunosuppressive factors like TGF-β and IL-10, which impairs NK cell recruitment and metabolic fitness [116, 119].

#### Human immunodeficiency virus

HIV, an RNA virus of the Retroviridae family, is the causative agent of acquired immune deficiency syndrome (AIDS) [160, 161]. It remains a global epidemic with no cure, affecting over 30 million people worldwide and posing a major health burden [162]. During HIV infection, NK cells undergo profound quantitative and qualitative alterations that collectively impair their antiviral capacity while paradoxically retaining partial protective functions [163]. Quantitatively, the total NK cell count expands transiently in acute infection but declines during chronic phases, with higher baseline NK cell frequencies predicting lower plasma viral loads and delayed CD4^+^ T cell depletion [164, 165]. Substantial subpopulation redistribution occurs, characterized by expansion of the dysfunctional CD56^−^CD16^+^ subset and depletion of cytotoxic CD56^dim^CD16^+^ and cytokine-producing CD56^bright^CD16^−^ populations. Notably, early anti-retroviral therapy (ART) preserves normal NK cell subset distributions, whereas delayed treatment perpetuates CD56^−^ subset expansion that correlates with impaired post-therapy immune reconstitution [164, 166]. Distinct functional subpopulations, including CD11b^+^CD57^−^CD161^+^Siglec-7^+^CD56^dim^CD16^+^, CXCR5^+^, and Siglec-9^+^CD56^dim^ NK cells, are associated with spontaneous viral control and reduced HIV DNA levels [167–169]. Specifically, high-dimensional mass cytometry (CyTOF) and machine learning identify the CD11b^+^CD57^−^CD161^+^ Siglec-7^+^CD56^dim^CD16^+^ subset as a mature, highly functional population that is enriched in elite controllers and inversely correlates with the viral reservoir size [167]. Follicular-homing CXCR5^+^ NK cells are distinguished by their capacity to migrate into B-cell follicles, which is a major, immunologically privileged reservoir of persistent replication-competent HIV [168]. Specifically, CXCR5 guides NK cells migration toward CXCL13, which is a chemokine secreted by follicular DC and stromal cells within the germinal centers [168, 170]. Once localized in the follicles by local IL-15 signaling, these NK cells upregulate activating receptors such as NKG2D and NKp44, enabling them to directly target and eliminate infected follicular helper T (Tfh) cells [168, 170, 171]. Furthermore, Siglec-9^+^CD56^dim^ NK cells exhibit a highly activated phenotype with superior cytotoxic potential against HIV-infected targets [169].

Immune checkpoint dysregulation further compromises NK cell functions during HIV infections through up-regulation of inhibitory receptors (TIGIT [172, 173], PD-1 [174], CD300a [175], and NKG2A [176]) and down-regulation of activating receptors (NCRs [177], NKG2D [178]). Specifically, TIGIT expression correlates positively with viral load and inversely with killing efficacy, whereas NKG2A upregulation on CD56^dim^ NK cells associates with advanced disease progression [172]. Despite these impairments, NK cells retain critical protective functions through direct cytotoxicity via perforin/granzyme secretion, Fas/FasL-mediated apoptosis, and chemokine production (CCL3/CCL5), as well as ADCC mediated by CD16 engagement with Fc regions of HIV-specific antibodies [179, 180].

Despite the global functional impairments observed during chronic HIV progression, a specialized subset of NK cells retains the ability to mount an adaptive, antigen-specific memory response against HIV-1 [43, 45, 181]. Strikingly, like the influenza-specific NK memory response, this mechanism relies heavily on the CD94/NKG2C receptor and its ligand HLA-E, as antibody blockade or genetic knockdown of NKG2C significantly abolishes these antigen-specific memory NK cell responses [43]. These cells recognize specific HLA-E-restricted nonameric peptides such as VLKYWWNLL from HIV-1 Env and Gag [43]. Upon re-encountering these cognate HIV antigens, the adaptive KLRG1^hi^α4β7^hi^NKG2C^+^ NK cell subset unleashes targeted and epitope-specific cytotoxicity, providing a crucial layer of antiviral defense that holds significant potential for future HIV-1 vaccine designs or cell-based therapeutics [43]. This suggest an expansion of NK cells with HLA-E-restricted nonameric peptides such as VLKYWWNLL may be a new NK cell therapy for HIV infection.

### Virus induced latent/reactivating infections

Latent and reactivating viral infections, predominantly exemplified by the herpesviridae family, establish a lifelong co-evolutionary dynamic with the host immune system [100, 182]. Unlike transient acute encounters, these viruses undergo periodic cycles of latency and reactivation, necessitating persistent and stringent immune surveillance [182]. For NK cells, this unrelenting cycle of antigenic exposure and inflammatory signaling drives profound phenotypic and functional remodeling, culminating in the generation of “adaptive” or “memory-like” NK cell subsets [39, 183]. These specialized subpopulations, characterized by extensive clonal-like expansion, exhibit superior targeted effector functions upon viral reactivation [39, 183]. Understanding NK cell dynamics in latent infections not only provides critical insights into the generation of innate immunological memory but also highlights the evolutionary compromises between viral persistence and host immune control.

#### Human cytomegalovirus

HCMV, a double-stranded DNA virus of the herpesviridae family [184], exhibits an age-dependent global seroprevalence ranging from 30% to 90% [185, 186]. HCMV infection serves as the canonical model for understanding immunological memory in the innate immune system, driving the epigenetic reprogramming and long-term memory-like responses of innate immune cells, particularly NK cells [40], and providing a classical framework for the concept of trained immunity [187]. A hallmark of the host immune response to HCMV is the robust, antigen-driven expansion of a specialized subset of adaptive-like NK cells, predominantly characterized by the CD56^dim^CD16^bright^NKG2C^+^ phenotype [188, 189]. Unlike canonical innate NK cell activation, the clonal-like expansion of HCMV-adaptive NK cells is strictly driven by specific viral antigens. During infection, HCMV employs the viral protein UL40 to provide nonameric peptides that stabilize the expression of host HLA-E molecules on the infected cell surface, a strategy initially evolved to evade NKG2A-mediated NK cell inhibition [190–192]. However, the adaptive NKG2C^+^ NK cell subset uniquely exploits this evasion mechanism [42, 193]. The activating receptor CD94/NKG2C specifically recognizes these UL40-derived polymorphic peptides presented by HLA-E [194]. Intriguingly, distinct UL40 peptide variants dictate differential activation avidities, which profoundly shape the inter-individual heterogeneity of the HCMV-induced memory NK cell repertoire and dictate their subsequent antiviral efficacy [42]. For instance, the canonical variant VMAPRTLIL strongly engages the inhibitory receptor NKG2A, effectively suppressing conventional NK cells [192]. In contrast, specific variant peptides, most notably VMAPRTLFL, display a significantly higher avidity for the activating receptor NKG2C. When presented on HLA-E, the VMAPRTLFL variant potently overcomes inhibitory thresholds, directly driving the robust activation, clonal expansion, and functional enhancement (e.g. elevated IFN-γ and TNF-α secretion) of NKG2C^+^ NK cells [42].

The functional significance of the adaptive NK cells extends beyond their expansion, where they provide a critical layer of protection by effectively limiting HCMV dissemination and controlling reactivation. Compared to their naive counterparts, HCMV-specific memory NK cells exhibit a “primed” state, characterized by rapid and robust polyfunctional responses—including massive IFN-γ production and potent degranulation—upon re-encountering the virus [3, 189]. This is particularly vital in immunocompromised settings, such as HSC or solid organ transplantation, where adaptive NK cells can bridge the temporal gap before T-cell recovery, significantly reducing the duration and severity of viremia [189, 195]. This human phenomenon finds its analog in the MCMV model, which serves as the gold standard for innate memory research. In C57BL/6 mice, the recognition of the viral MHC-I homolog m157 by the activating receptor Ly49H (the functional ortholog of human NKG2C) triggers a massive, antigen-specific clonal expansion of NK cells [8, 11]. Much like the human model, these m157-reactive NK cells survive long-term as memory populations. Upon secondary MCMV challenge, these memory NK cells execute a swift recall response that dramatically lowers viral titers in the spleen and liver, demonstrating that NK cell-mediated memory is a conserved and non-redundant evolutionary strategy for controlling persistent herpesvirus infections [11, 39].

Moreover, in humans, HCMV seropositivity is strongly correlated with elevated circulating levels of monocyte-derived IL-12 [193]. Rather than inducing a non-specific, pan-activation of the entire NK cell compartment, IL-12 acts in strict synergy with the specific engagement of NKG2C by viral peptide-presenting HLA-E [193]. Mechanistically, this dual-signal convergence uniquely upregulates CD25 (the high-affinity IL-2 receptor α chain) exclusively on the NKG2C^+^ subset [193]. This critical receptor reprogramming sensitizes these specific cells to T cell-derived IL-2, fueling their selective, clonal-like expansion and long-term survival [42, 193]. Consequently, HCMV infection rarely provokes a global quantitative increase in the total peripheral NK cell pool; instead, it drives a profound qualitative skewing of the receptor repertoire [7]. This manifests as a persistent and disproportionate amplification of terminally differentiated NKG2C^+^CD57^+^ memory-like NK cells, concomitant with a stable absolute NK cell count but a proportional contraction of conventional NKG2A^+^ subsets [7, 195].

Following antigen and cytokine stimulation, HCMV-adapted NK cells undergo profound epigenetic remodeling, distinguishing them functionally from conventional NK cells. A critical phenotypic signature of these mature NKG2C^+^ cells is the epigenetic silencing of the adaptor protein FcεR1γ (resulting in a FcεR1γ^−^ profile) and transcription factors such as PLZF via DNA hypermethylation [189, 196]. This remodeling rewires their intracellular signaling pathways, endowing the CD57^+^FcεR1γ^−^NKG2C^+^ subset with significantly enhanced capacity for ADCC [196, 197]. Functionally, these adaptive NK cells exhibit superior degranulation against target cells compared to canonical subsets [189, 194]. This cytotoxicity is further potentiated when CD16 and NKG2C are co-stimulated, inducing more robust degranulation than CD16 ligation alone [197], which is also supported by the finding that anti-NKG2C/IL-15/anti-CD33 killer engager could selectively drive the expansion of NKG2C^+^ NK cells in both peripheral and iPSC derived NK cells [198]. Importantly, HCMV-specific sera and purified viral particles trigger higher functional efficacy in NKG2C^+^ NK cells relative to NKG2C^−^ subsets [199], highlighting their highly specialized virus-specific responsiveness [42].

The clinical relevance of HCMV-adapted NK cells is most prominently observed in hematopoietic stem cell transplant (HSCT) recipients [200, 201]. Following HCMV reactivation post-HSCT, circulating NK cell counts undergo a rapid 10-fold increase, whereas NK cell numbers remain stable in non-reactivated individuals [201]. During this dynamic process, immature CD56^bright^ NK cells reach their expansion peak at 6 months, while the mature CD56^dim^ NKG2C^+^ subsets sustain prolonged proliferation [188]. Notably, a study of patients receiving T-cell-depleted allogeneic HSCT demonstrated that 92% of those with HCMV reactivation developed elevated frequencies of NKG2C^+^ NK cells [200]. Crucially, the emergence of these adaptive NK cells exhibits a potent graft-versus-leukemia effect *in vivo*. Clinical cohorts have corroborated that this HCMV-driven repertoire skewing is strongly associated with a significantly reduced risk of leukemia relapse and improved survival following transplantation [202, 203]. Ultimately, such profound clinical benefits, coupled with their robust and long-lasting expansion, underscores the potential of exploiting HCMV-driven adaptive NK cells as a therapeutic cellular platform for combating refractory viral infections and malignancies in immunocompromised populations [204].

The clinical and translational significance of these adaptive NK cell dynamics is most prominently manifested during HCMV reactivation in allogeneic HSCT [203, 205]. Following HSCT, donor-derived NK cells are the first lymphoid lineage to reconstitute, and their rapid recovery (typically within 3–4 weeks) is crucial to control early post-transplant HCMV replication [206]. In these immunocompromised hosts, HCMV reactivation drives a profound expansion of the mature NKG2C^+^CD57^+^ adaptive NK cells [201, 207]. These reconstituted cells provide rapid and targeted protective immunity through the afore-mentioned NKG2C/HLA-E recognition axis [208, 209]. Exploiting this innate antiviral potency, clinical trials are actively investigating the adoptive transfer of either donor-derived activated NK cells or CIML NK cells as safe, pre-emptive therapies to control drug-refractory HCMV reactivations post-transplant without provoking GVHD [46, 210].

#### Epstein–Barr virus

EBV is a ubiquitous γ-herpesvirus mainly transmitted via saliva, which establishes lifelong latent infection in over 95% of the global population typically without clinical manifestations, yet can induce infectious mononucleosis (IM) in adolescents and is linked to multiple hematologic and epithelial malignancies, particularly in individuals with immunodeficiency [211, 212]. NK cells play a multifaceted and non-redundant role in mediating resistance against EBV infection across all phases of the viral life cycle. During acute primary EBV infection, prompt NK-cell expansion restricts lytic replication, whereas experimental depletion of NK cells in humanized mouse models leads to a 10- to 100-fold increase in EBV viral loads in both blood and spleen, a significant elevation in the spleen-to-body weight ratio, and a drastically higher incidence of macroscopic lymphomas within six weeks [213]. However, in chronic active EBV (CAEBV) disease, progressive NK lymphopenia and impaired effector functions strongly correlate with persistent viremia, systemic lymphoproliferation, and poor clinical prognosis [54, 214]. Extensive clinical registries reveal that ~33% of CAEBV patients present with severely reduced circulating NK cell counts (typically <90 cells/µl or <3% of total lymphocyte) [54].

During primary EBV infection, the early-differentiated NK cell subset CD56^dim^NKG2A^+^KIR^−^CD57^−^ undergoes rapid and specific expansion, which is predominantly driven by direct stimulation from lytic EBV infection [214]. Unlike the peptide-specific memory responses observed in HCMV, this expansion is not driven by a singular viral antigen, but is predominantly triggered by the globally altered surface profile of B cells undergoing lytic EBV replication [214]. Specifically, early lytic viral proteins (such as the master transcription factor BZLF1) orchestrate a dramatic remodeling of the host B-cell surface, characterized by the upregulation of activating stress ligands (e.g. MICB, ULBPs) alongside the concurrent downregulation of classical MHC-I and HLA-E molecules, which releases the inhibitory “brakes” on NK cells while engaging their activating receptors (such as NKG2D) to pull the “triggers” for proliferation [213, 215]. Consequently, the proliferation of this subset is highly dependent on the expression of EBV lytic antigens: the frequency of Ki-67^+^ proliferating cells within this subset is significantly elevated during acute IM, and positively correlates with EBV loads in peripheral blood mononuclear cells; in contrast, infection with a BZLF1-knockout EBV mutant, which lacks the master transcription factor required to trigger the lytic cascade, fails to induce the immunostimulatory surface alterations on infected B cells, resulting in a markedly reduced proliferation of this NK subset [213, 214]. Meanwhile, this NK cell subset exerts direct cytotoxicity against EBV-infected B cells via the “missing-self” and “induced-self” recognition models: for missing-self recognition, EBV lytic infection induces the downregulation of classical HLA class I (HLA-A/B/C) and non-classical HLA-E molecules on the surface of infected B cells, which abrogates the inhibitory signals transduced by NKG2A and KIRs, thus lowering the activation threshold of NK cells [214, 216]; for induced-self recognition, lytic EBV infection upregulates the stress-induced ligands ULBP1 and CD112 on infected B cells, which engage the activating receptors NKG2D and DNAM-1 on NK cells, respectively. This has been confirmed to functionally trigger NK cell activation via specific antibody blocking assays, ultimately promoting the release of lytic granules and the induction of apoptosis in target cells [213, 214, 216]. Notably, despite NKG2A functioning as a canonical inhibitory receptor, NKG2A^+^ NK cells demonstrate superior cytotoxicity against EBV-transformed lymphoblastoid cell lines (LCLs) due to these NK cells are highly “licensed” during development, endowing them with intrinsically superior cytotoxic potential [217]. Besides, specific EBV-derived peptides uniquely bind to host HLA-E molecules on infected cells but form altered peptide-HLA-E complexes that fail to effectively engage the NKG2A receptor, disrupting the inhibitory NKG2A/HLA-E signaling axis and fully unleashing the potent cytotoxic capacity of these educated NK cells [217, 218]. Besides, this loss of NKG2A-mediated inhibition might tip the competitive balance toward the activating CD94/NKG2C receptor, which remains capable of binding these altered peptide-HLA-E complexes to trigger positive activating signals [219, 220].

During the expansion phase of acute IM, NK cells also exert immunoregulatory functions by eliminating overactivated CD8^+^ T cells, preventing excessive immune pathology [213]. Mechanistically, as CD8^+^ T cells undergo massive, antigen-driven proliferation, they experience significant cellular stress and subsequently upregulate “induced-self” molecules, particularly NKG2D ligands (e.g. MICA/B and ULBPs), alongside the death receptor Fas (CD95) [221, 222]. NK cells sense these stress signatures and actively eliminated the hyperactivated T cell via NKG2D-mediated perforin/granzyme degranulation and Fas/FasL-dependent apoptosis [221, 222]. This rheostat-like regulatory mechanism is essential for resolving the acute inflammatory phase of IM and restoring overall immune homeostasis [223]. During latent EBV infection, although latently infected B cells evade NK cell recognition via high HLA-I expression and viral evasion strategies (e.g. miR-BART2-5p-mediated downregulation of MICB [224]), NK cells retain the capacity to target reactivated lytic cells or EBV-associated malignant cells, where HLA-I loss and upregulated NKG2DLs sensitize targets to NK cell-mediated killing [225, 226]. Additionally, NK cells mediate ADCC against lytic EBV-infected cells expressing gp350/220, albeit at moderate efficacy due to EBV’s suppression of functional antibody responses [227, 228]. Despite the robust NK cell-mediated surveillance during acute lytic replication, EBV successfully establishes lifelong latency by driving infected B cells into a highly restricted resting state [229, 230]. In this latent reservoir, the virus maintains physiological HLA class I expression and silences immunogenic lytic proteins, thereby effectively stripping away the molecular signatures required for both “missing-self” and “induced-self” NK cell recognition [229, 230].

## Mechanisms of viral subversion of Nk cell surveillance

**Figure 7:**
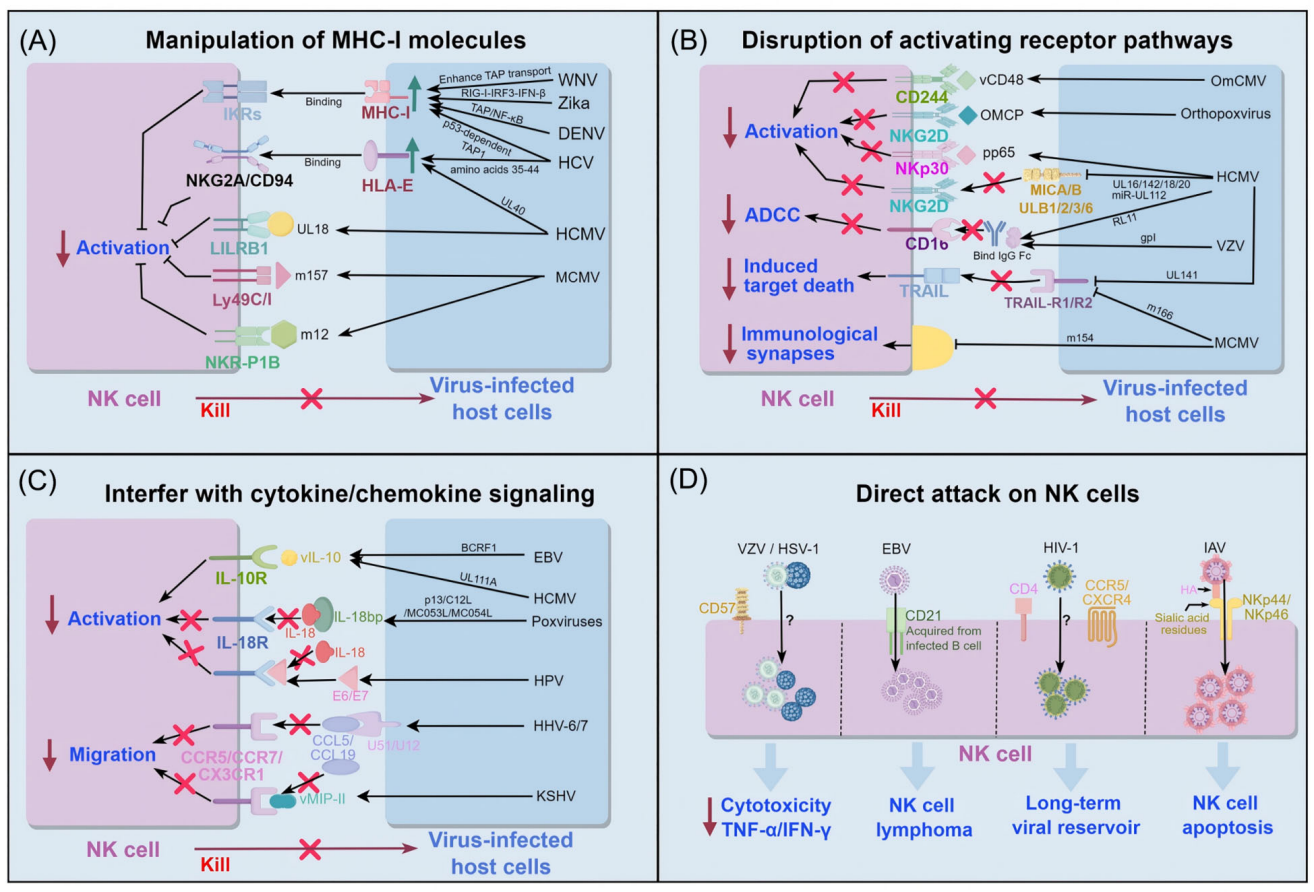
Strategies of virus to evade NK cell immunosurveillance. Viruses employ diverse and complex mechanisms to evade NK cells. (A) Manipulation of MHC-I molecules: to balance T-cell evasion with NK-cell avoidance, viruses utilize “molecular mimicry” and stabilized inhibitory signaling. Beyond downregulating classical MHC-I to escape CD8^+^ T cells, viruses deploy specific peptides to stabilize HLA-E expression, while HCMV UL40-derived peptides successfully engage CD94/NKG2A to maintain this inhibitory checkpoint, the SARS-CoV-2 Nsp13-derived peptide (VMPLSAPTL) represents a molecular mismatch that fails to trigger inhibitory signaling, thereby abrogating inhibition and permitting target cell lysis. Additionally, viral MHC-I homologs (e.g. UL18, m157) engage inhibitory receptors (LILRB1, Ly49C/I) with high affinity to provide “false-self” signals that shield infected cells from NK cell-mediated lysis. (B) Disruption of activating receptor pathways: viruses employ targeted sabotage of activating signals through multiple layers, including secreting soluble decoys (e.g. OMCP, vCD48) to competitively block receptors such as NKG2D and 2B4, disrupting ligand surface expression via intracellular retention in the ER/Golgi (e.g. UL16, UL142), lysosomal degradation (e.g. US18/20), or microRNA-mediated translational repression (e.g. miR-UL112), and impeding ADCC and synapse stability through FcγR mimics or the degradation of adhesion molecules. (C) Interfer with cytokine/chemokine signaling: viruses secret “virokines” or “viroceptors” to disrupt the local immune microenvironment. Viral homologs of IL-10 (vIL-10) and IL-18 binding proteins (vIL-18bp) neutralize pro-inflammatory signals, while viral chemokines (e.g. vMIP-II) or receptor mimics (e.g. U51, U12) interfere with NK cell chemotaxis and tissue homing by sequestering ligands such as CCL5 and CCL19. (D) Direct attack on NK cells: herpesviruses (e.g. VZV, HSV-1) and HIV-1 can directly infect NK cells, establishing long-term viral reservoirs or impairing cytokine secretion (IFN-γ, TNF-α). Furthermore, IAV utilizes hemagglutinin (HA) to bind NKp44/NKp46, triggering caspase-dependent apoptosis of the effector cells.

The continuous co-evolution between human hosts and viral pathogens has precipitated a complex immunological arms race [231]. While NK cells act as a formidable first line of defense, viruses have concomitantly evolved highly sophisticated, multifaceted evasion strategies to bypass this innate immune surveillance [3, 232]. To establish successful primary infection, facilitate systemic dissemination, and maintain long-term latency, it is an evolutionary imperative for viruses to profoundly subvert NK cell-mediated recognition and cytolytic functions [3, 233]. This section systematically outlines the four primary paradigms of viral evasion against NK cells. These strategies range from the direct infection and functional paralysis of NK cells themselves to the targeted sabotage of the host’s local immune microenvironment via interference with critical cytokine and chemokine signaling networks. Furthermore, viruses extensively remodel the surface of infected cells, employing molecular decoys and intracellular retention pathways to disrupt the engagement of NK cell activating receptors. Simultaneously, they dynamically manipulate the expression of classical and non-classical MHC-I molecules to strategically exploit inhibitory signaling and escape “missing-self” recognition. Collectively, mapping these intricate viral countermeasures not only deepens our understanding of viral pathogenesis but also exposes specific molecular targets that can be developed for next-generation of NK cell-directed immunotherapies (Fig. 7).

### Manipulation of MHC-I molecules

Viruses precisely modulate the expression of classical and non-classical MHC-I molecules to evade NK cell inhibitory recognition [234]. Although some viruses down-regulate surface MHC-I to avoid CD8^+^ T cell recognition, consequently exposing themselves to NK-cell “missing-self” detection [235], other pathogens, particularly flaviviruses, employ the exact opposite strategy [236]. To shield themselves from NK-cell cytolysis during acute infection, these viruses have evolved to upregulate host cell-surface MHC-I, thereby delivering enhanced inhibitory signals through NK-cell receptors such as LIRs or KIRs [1, 236]. West Nile virus enhances peptide transport into the endoplasmic reticulum via TAP, thereby increasing MHC-I surface expression without altering TAP protein levels [237]. Similarly, expression of dengue virus replicon in K562 or THP1 cells induces MHC-I upregulation and aggregation on the cell membrane through both TAP-dependent and -independent mechanisms, which further contribute to the higher binding of low-affinity inhibitory receptor on NK cells (e.g. LIR1) [238]. Zika virus exploits the RIG-I-IRF3 pathway to induce IFN-β-mediated MHC-I upregulation, effectively escaping NK detection despite minimal induction of activating ligands [239]. Moreover, HCV core protein indirectly upregulates MHC-I via p53-dependent TAP1 transcription, impairing NK cytotoxicity while preserving cytotoxic T lymphocyte recognition [240]. Although this upregulation theoretically increases susceptibility to adaptive immune attack, HCV actively resolves this paradox by selectively degrading classical antigen-presenting molecules. Specifically, the HCV core protein physically interferes with the host intramembrane protease signal peptide peptidase (SPP), which is normally required for classical MHC-I (such as HLA-A) maturation [241]. By blocking SPP–HLA-A interactions, the core protein induces the accumulation of immature HLA-A molecules, which are subsequently targeted by the E3 ubiquitin ligase HRD1 for proteasomal degradation, thereby successfully impairing antigen presentation to CD8^+^ T cells [241]. Thus, HCV employs a dual-evasion strategy: upregulating non-classical or “empty” MHC-I to engage inhibitory NK receptors while simultaneously degrading mature classical MHC-I via the SPP/HRD1 pathway to escape CD8^+^ T-cell surveillance [240].

For non-classical MHC-I modulation, viruses exploit the NKG2A/CD94 inhibitory pathway by providing peptides that stabilize HLA-E (in humans) or Qa-1 (in mice) [190, 242]. HCMV UL40 encodes a peptide that mimics the HLA-E leader sequence, stabilizing HLA-E expression and engaging the inhibitory NKG2A/CD94 receptor [190, 192]. Similarly, a peptide derived from HCV core protein (amino acids 35–44) binds to and stabilizes HLA-E, enhancing interaction with the inhibitory NKG2A receptor [243]. Besides, rodent herpesvirus Peru (RHVP) could encode pQa-1, a viral mimic of Qa-1, directly engages the inhibitory NKG2A/CD94 complex and circumvent NK cell activation [244].

Several viruses encode MHC-I-like molecules that directly engage NK cell receptors [3]. For example, HCMV UL18, an MHC-I homolog, binds with high affinity to the inhibitory receptor LILRB1 and with lower affinity to the activating receptor NKG2C [28, 245], leading to diminished NK cytotoxicity. MCMV m157 could bind to the inhibitory receptors Ly49C/I on NK cells in 129/J mice, promoting host susceptibility to infection [246, 247]. MCMV m12 functions as a molecular decoy to preferentially bind the inhibitory NKR-P1B receptor on NK cells via a unique “polar claw” docking model and deliver dominant inhibitory signals that shield infected cells from immune clearance [248]. Besides, rat cytomegalovirus (RCMV) can encode the viral C-type lectin-like protein RCTL, which also acts as a molecular decoy to directly engage the inhibitory NKR-P1B receptor on NK cells [249].

### Disruption of activating receptor pathways

Viruses produce decoy receptors or soluble inhibitors that directly engage and block activating receptors on NK cells [3]. For instance, owl monkey cytomegalovirus (OmCMV) encodes a soluble viral CD48 (vCD48) via its A43 gene, which antagonizes the SLAM family receptor 2B4 (CD244) on NK cells [250]. HCMV pp65 protein binds to the NKp30 receptor, reducing NK cell cytotoxicity [251]. Orthopoxviruses, such as cowpox virus and monkeypox virus, secrete the orthopoxvirus MHC-I-like protein (OMCP), a soluble NKG2D ligand that competitively binds NKG2D and inhibits NK cell activity [252, 253].

A prevalent viral strategy involves the degradation, retention, or downregulation of ligands for activating receptors such as NKG2D, DNAM-1, and SLAM family members [3]. To evade NKG2D recognition, HCMV employs multiple ways to reduce the expression of its ligands: UL16 and UL142 interacts with MICB, ULBP1, ULBP2, ULBP6, ULBP3, and MICA, retaining them in the ER/Golgi, preventing them from trafficking to the surface and protecting the infected cells from NK cell-mediated cytotoxicity [254]; US18 and US20 mediate lysosomal degradation of MICA, MICB, and ULBP1, 2, 3, 6 [255]; and the microRNA miR-UL112 targets the 3'UTR of MICB mRNA to suppress its translation [224, 256]. Mouse cytomegalovirus (MCMV) uses m138, m145, m152, and m155 to down-regulate the NKG2D ligands RAE-1, MULT-1, and H60 [3]. In addition, some other viruses also employ similar strategies to suppress the activating ligands for NKG2D. For example, HHV-7 U21 drives the degradation of MICA and MIC by redirecting them from the trans-Golgi network to lysosomes for proteolysis and thereby preventing surface translocation [257]; Kaposi’s sarcoma-associated herpesvirus (KSHV) not only encodes miR-K12-7 to bind the distinct adjacent sites within the MICB 3'UTR to repress its translation [224], but also generate K5 E3 ubiquitin ligase to mediate MICA degradation [257]; adenovirus 5 E3/19K sequesters MICA and MICB in the ER [258]; and HIV-1 Nef inhibits the surface expression of MICA, ULBP1, and ULBP2 [259]. To disrupt DNAM-1 signaling, HCMV UL141 and MCMV m20.1 degrade the ligand CD112 and CD155 [260–262], while HSV-2 and pseudorabies virus glycoprotein D (gD) downregulates CD112 [263]. For SLAM family receptor evasion, MCMV m154 drives proteasomal and lysosomal degradation of CD48 without altering its transcription, which is the high-affinity ligand for NK cell-activating receptor 2B4, to evade NK cell-mediated immune surveillance [264].

Viruses impede the final execution of NK cell cytotoxicity by targeting FcγR-mediated ADCC, TRAIL-induced apoptosis, and immunological synapse formation [3]. To disrupt ADCC, herpesviruses encode Fcγ receptor mimics: HSV-1/2 glycoprotein E/gI complex, varicella zoster virus (VZV) glycoprotein I (gpI), HCMV RL11 and UL119-118, and rhesus CMV (RhCMV) RL11 bind the Fc portion of IgG, preventing antibody-mediated NK cell degranulation [265]. To inhibit TRAIL-mediated apoptosis, HCMV UL141 binds to TRAIL death receptors (TRAIL-R1/R2), preventing their surface expression [266], and MCMV m166 restricts the cell-surface expression of TRAIL death receptors via an incompletely understood post-transcriptional mechanism that likely avoids direct physical interaction with the receptors [265, 267]. Furthermore, viruses disrupt the formation of stable immunological synapses via remodeling the actin cytoskeleton in target cells. For example, HCMV pUL135 recruits WAVE2 to remodel actin cytoskeleton [268], and UL148 causes intracellular retention of CD58 (LFA-3), which is an adhesion molecule that is critical for effector cell co-stimulation and immune synapse formation [269]. Furthermore, cytomegaloviruses employ distinct cellular strategies to subvert the CD48-2B4 co-signaling axis, which is critical for NK-cell conjugate formation and immunological synapse stability [250, 264]. MCMV encodes the mucin-like early protein m154, which utilizes its cytoplasmic dileucine-like “DD” motif to hijack the host adaptor protein-1 (AP-1) sorting complex [264, 270]. This perturbation redirects the SLAM family member CD48 (the natural ligand for 2B4) to lysosomal proteolytic degradation, thereby blunting NK cell-mediated cytotoxicity [264, 270]. In contrast, OmCMV targets the same axis extracellularly by encoding A43, a highly glycosylated viral homolog of CD48 (vCD48) [250]. Following proteolytic cleavage near its membrane-proximal region, A43 is released as a soluble decoy receptor that binds host 2B4 with exceptionally high affinity and slow dissociation rates, effectively masking 2B4 and preventing stable immune synapse establishment [250].

### Interference with cytokine and chemokine signaling

Viruses encode homologs, decoy receptors, or sequestering factors to disrupt cytokine and chemokine signaling pathways crucial for NK cell activation and recruitment [3]. To suppress cytokine signals, HCMV UL111A and EBV BCRF1 encode viral IL-10 (vIL-10): EBV BCRF1 directly protects newly infected B cells from NK cell mediated lysis [271], whereas HCMV UL111A indirectly blunts NK activation by inhibiting dendritic cell maturation and subsequent IL-12/IL-15 secretion [272, 273]. Poxviruses, including ectromelia virus (p13), vaccinia virus (C12L), and molluscum contagiosum virus (MC053L/MC054L), produce viral IL-18 binding proteins (vIL-18BP) that bind and neutralize IL-18, thereby inhibiting IFN-γ production by NK cells [274–276]. HPV employs its E6 and E7 oncoproteins to competitively bind the IL-18 receptor alpha (IL-18Rα) on NK cells, reducing IL-18-dependent IFN-γ production [277]. To interfere with chemokine signaling, human herpesviruses 6 and 7 (HHV-6/7) encode U51 and U12 proteins that mimic chemokine receptors (e.g. CCR5, CCR7), sequestering ligands such as CCL5 and CCL19 and impeding NK cell migration [278–281]. KSHV vMIP-II acts as a viral chemokine that competitively inhibits receptors such as CX3CR1 and CCR5, blocking NK cell chemotaxis [282].

### Direct attack on NK cells

Several viruses have evolved the capacity to directly infect NK cells, thereby compromising their antiviral effector functions [3]. Herpesviruses, including VZV, herpes simplex virus 1 (HSV-1), and EBV, can productively infect human NK cells [283, 284]. VZV preferentially infects mature CD56^dim^CD57^+^ NK subsets via cell-to-cell contact, leading to impaired degranulation and reduced production of TNF-α and IFN-γ [283, 284]. *In vitro* evidence also proves that HSV-1 can infect and express viral proteins in human NK cells, resulting in a 14-fold reduction of IFN-γ secretion [283]. EBV, which primarily targets B cells via the CD21 receptor, can also enter NK cells after NK cells acquire CD21 expression during the formation of an immunological synapse with infected B cells, and persistent infection may lead to NK cell malignancies such as nasal-type NK lymphoma [285]. Retroviruses such as HIV-1 can infect proliferating NK cells, which upregulate CD4 and coreceptors (CCR5/CXCR4) in response to cytokine-driven activation, thereby becoming susceptible to productive infection and serving as a long-term viral reservoir [286–288]. Furthermore, RNA viruses such as IAV utilize their HA surface glycoproteins to bind sialic acid residues on the activating receptors NKp44 and NKp46, facilitating entry into NK cells [65, 66, 70], which leads to caspase-3-dependent apoptosis of infected NK cells *in vitro*, representing an apoptosis-based evasion mechanism [65]. Although studies report direct antibody blockade of NKp46 or NKp44 can prevent viral entry and protect NK cells from caspase-3-dependent apoptosis [65], this approach carries the critical drawback of compromising NK cell activation and reducing natural cytotoxicity against infected cells. To bypass this limitation, soluble chimeric NKp46-Ig or NKp44-Ig fusion proteins serve as a superior alternative. By acting as molecular decoys that competitively bind viral HA, these soluble receptors neutralize the virus without blocking receptors on NK cells; moreover, their IgG-Fc fusion domain can actively trigger NK cell activation via ADCC, establishing a highly elegant therapeutic strategy [65, 70].

Overall, the evolutionary arms race between viruses and host immunity has driven viruses to evolve an array of proteins and peptides that disrupt NK cell immunosurveillance. To provide a clear mechanistic overview, we have comprehensively summarized the diverse viral factors currently known to directly modulate, mimic, or hijack NK cell activating and inhibitory receptor signaling pathways in Table 2. Elucidating these molecular evasion strategies not only clarifies viral pathogenesis but also reveals actionable targets for next-generation NK cell immunotherapies.

**Table 2:** Viral peptides and proteins modulating NK cell activating or inhibitory receptor signaling.

Virus	Viral factor (Type)	Origin gene	Targets on NK cells	Influence on NK cells	Ref.
HCMV	**VMAPRTLIL** *(Peptide)*	***UL40***	CD94/NKG2A (via HLA-E)	**Inhibitory:** Mimics the host MHC-I leader peptide, stabilizing HLA-E cell-surface expression to deliver dominant inhibitory signals to NKG2A^+^ NK cells to evade cytolysis.	[42, 192]
HCMV	**VMAPRTLFL** *(Peptide)*	***UL40***	CD94/NKG2C (via HLA-E)	**Activating:** Binds HLA-E with high affinity, driving clonal expansion and differentiation of adaptive/memory-like (CD57^+^ NKG2C^+^) NK cells.	[42]
HCMV	**UL18** *(Protein)*	***UL18***	LILRB1 (LIR-1)	**Inhibitory Decoy:** Binds inhibitory LILRB1 with >1000-fold higher affinity than host classical MHC-I, delivering dominant “false-self” inhibitory signals.	[28]
HIV-1	**VLKYWWNLL** *(Peptide)*	***Env/Gag***	CD94/NKG2C (via HLA-E)	**Activating:** Promotes epitope-specific memory recall and cytotoxicity in KLRG1^hi^ α4β7^hi^ memory NK cells.	[43]
HIV-1	**Vpu** *(Protein)*	***Vpu***	2B4 & NTB-A (via CD48 & NTB-A downregulation)	**Inhibitory (Trafficking Block):** Downmodulates CD48 and NTB-A by disrupting their glycosylation, impairing NK degranulation and CD16-mediated ADCC.	[313, 425]
IAV	**TMDSNTLEL** *(Peptide)*	**NP (Nucleoprotein)**	CD94/NKG2C (via HLA-E)	**Activating:** Triggers antigen-driven recall and robust secondary cytotoxic responses of memory-like CD94/NKG2C^+^ NK cells.	[43]
HCV	**YLLPRRGPRL** *(Peptide)*	**Core (aa 35–44)**	CD94/NKG2A (via HLA-E)	**Inhibitory:** Core-derived peptide stabilizes surface HLA-E, delivering inhibitory signals that protect infected hepatocytes from cytolysis.	[243]
EBV	**GGDPHLPTL** *(Peptide)*	***LMP-1***	CD94/NKG2A (via HLA-E)	**Inhibitory:** Strongly suppresses CD94/NKG2A^+^ NK cells, promoting viral latency, immune evasion, and PTLD development.	[220]
EBV	**SQAPLPCVL** *(Peptide)*	***BZLF1***	CD94/NKG2A (via HLA-E)	**Abrogates Inhibition (Activating):** Acts as an altered peptide ligand that fails to engage CD94/NKG2A, facilitating T and NK-cell lytic clearance.	[218, 220]
RCMV	**RCTL** *(Protein)*	***RCTL***	NKR-P1B (inhibitory)	**Inhibitory Decoy:** Acts as a C-type lectin-like mimic of host Clr-b to bind inhibitory NKR-P1B, bypassing “missing-self” NK surveillance.	[249]
MCMV	**m157** *(Protein)*	***m157***	Ly49H (activating)/Ly49C/I (inhibitory)	**Dual-action Decoy:** Binds activating Ly49H (in C57BL/6) to drive memory NK cell expansion, but binds inhibitory Ly49C/I (in 129/J) to deliver inhibitory signals.	[426, 427]
MCMV	**m12** *(Protein)*	***m12***	NKR-P1B (inhibitory)/NKR-P1C (activating)	**Dual-action Decoy:** Binds NKR-P1B to shield targets, while interacting with NKR-P1C to drive clonal expansion of liver-resident NK/ILC1s.	[248, 428]
MCMV	**m154** *(Protein)*	***m154***	2B4 (via host CD48 degradation)	**Inhibitory (Lysosomal Degradation):** Hijacks host AP-1 complex via its “DD” motif, routing CD48 (the ligand for activating 2B4) to lysosomes.	[264, 270]
OmCMV	**A43** *(Protein)*	***A43***	2B4 (CD244)	**Inhibitory Decoy:** Secretes a soluble CD48 viral homolog (vCD48) that acts as a high-affinity decoy, masking host 2B4 and disrupting NK synapse.	[250]
SARS-CoV-2	**VMPLSAPTL** *(Peptide)*	***Nsp13***	CD94/NKG2A (via HLA-E)	**Abrogates Inhibition (Activating):** Stabilizes HLA-E but fails to engage CD94/NKG2A due to steric mismatch, permitting target cell lysis.	[92]
SARS-CoV-2	**Nsp1** *(Protein)*	***Nsp1***	NKG2D (via host NKG2D-L downregulation)	**Inhibitory (Translation Shut-off):** Blocks host translation and protein synthesis, resulting in downregulated surface MICA/B and ULBPs.	[429]
SARS-CoV-2	**Spike 1** *(Protein)*	***Spike 1***	CD94/NKG2A (via HLA-E upregulation)	**Inhibitory (Indirect Upregulation):** Contacts lung epithelial cells to indirectly upregulate HLA-E, delivering potent CD94/NKG2A inhibitory signals.	[430]
HBV	**HBeAg** *(Protein)*	***HBeAg***	CD94/NKG2A (via Treg IL-10 upregulation)	**Inhibitory (Indirect Exhaustion):** Stimulates regulatory T cells (Tregs) to secrete IL-10, upregulating NKG2A and driving NK-cell exhaustion.	[116]

## NK cell-based antiviral immunotherapies

Viruses' ability to evade innate immunity and induce functional exhaustion underscores the urgent need for novel therapies. NK cells represent a promising platform for next-generation “off-the-shelf” antiviral immunotherapies, owing to their intrinsic antiviral activity, MHC-independent recognition, and superior safety profile with reduced GvHD and CRS risks [289–291]. To overcome viral immune evasion mechanisms and restore robust immune clearance, a wide range of NK cell-based strategies are being intensively investigated in preclinical and translational studies [1, 290]. Here, we outline key therapeutic approaches to unlock the full antiviral potential of NK cells and enable effective immunotherapeutic strategies for chronic viral infections (Table 3).

**Table 3:** Comprehensive summary of emerging NK cell-based antiviral immunotherapies.

Strategies	Specific agent/intervention	Target virus (Antigen)	Targets on NK cells	Functional outcomes	Current stage	Ref.
Checkpoint inhibitors & mAbs	Monalizumab (Humanized IgG4 anti-NKG2A)	HCV, HIV, HCMV, SARS-CoV-2	NKG2A/CD94	Disrupts NKG2A/HLA-E interaction; unleashes missing-self recognition, restores robust degranulation and IFN-γ secretion.	Pre-clinical/Clinical (for viral-associated malignancies)	[93, 295, 431–433]
	Lirilumab (Anti-KIRs)	HPV + malignancies	KIR2DL1/2/3	Abrogates suppressive interaction with HLA-C; promotes NF-κB disinhibition and restores cytolysis against infected cells.	Pre-clinical/Clinical (for viral-associated malignancies)	[298, 434]
	Sabatolimab (Anti-TIM-3)	HIV, HCV	TIM-3	Blocks TIM-3/Galectin-9 binding; mitigates chronic antigen-induced exhaustion and reverses NK cell hyporesponsiveness.	Pre-clinical/Clinical program terminated (Novartis, 2023)	[330, 435–437]
	Anti-TIGIT + IL-21	Chronic HBV	TIGIT & IL-21R	Synergistically relieves IFN-γ inhibition on CD56^dim^ subsets; promotes CD16/NKG2D expression and accelerates viral clearance.	Pre-clinical (Mouse)	[117, 332–335]
	Anti-CD16-based BiKEs	HIV (gp160), HBV (PreS1)	CD16 (FcγRIIIa)	Physically bridges CD16 to viral antigens; overrides KIR-HLA-I inhibitory signals to direct highly specific ADCC.	Pre-clinical	[317–320]
	IL-15 integrated TriKEs	HIV, HCMV	CD16 & IL-15R	Dual-targeting combined with localized IL-15 delivery; drives profound NK cell expansion, survival, and *in vivo* persistence.	Pre-clinical	[321–325]
CAR-NK cells	SARS-CoV-2 CAR-NK (e.g. S309 scFv/H84T-BanLec)	SARS-CoV-2 (Spike protein/glycans)	Intracellular DAP12, 2B4, DNAM-1	Bypasses viral evasion mechanisms; directly kills infected cells and suppresses pseudo-virus replication *in vitro*.	Pre-clinical	[357–359, 438–441]
	Universal HIV CAR-NK	HIV (Diverse gp160 epitopes)	Engineered scFv + Costimulatory domains	Utilizes DNP-conjugated antibodies to target highly mutable HIV variants; enables elimination of diverse subtype-infected cells.	Pre-clinical	[163, 360, 361]
	HBV/EBV CAR-NK	HBV (HBsAg/PreS1), EBV (gp350/LMPs)	Engineered scFv + Costimulatory domains	Exerts potent cytotoxicity against infected hepatocytes (reduces HBV cccDNA) and treats EBV-related lymphoproliferative disorders.	Pre-clinical	[362, 363, 365, 366]
Small molecule compounds	Selgantolimod (GS-9688) (TLR8 Agonist)	Chronic HBV	IL-12R & IL-18R (indirectly via TLR8 activation on hepatic mononuclear phagocytes)	Stimulates hepatic mononuclear phagocytes to secrete IL-12 and IL-18; breaks intrahepatic tolerance, restores NK cell IFN-γ production, and promotes hepatocyte lysis.	Phase II Clinical (e.g. NCT03491553)	[380, 381]
	APG-1387 (SMAC analog/IAP antagonist)	Chronic HBV, HBV-positive HCC	cIAP1, cIAP2, and XIAP (on target cell level)	Sensitizes infected hepatocytes to TNF-α-mediated apoptosis; expands the liver NK cell population and enhances innate liver immunity.	Phase I/II Clinical	[382]
	AZD5582 (SMAC analog/IAP antagonist)	HIV-1 (Latent CD4⁺ T cells)	cIAP1, cIAP2, and XIAP (on target cell level)	Reverses HIV-1 latency via the non-canonical NF-κB pathway; degrades XIAP/cIAPs to release caspases, lowering target cell apoptotic threshold and bypassing Nef-mediated cytolytic resistance.	Pre-clinical	[382–384]
	Vorinostat, Chidamide (CS055) (HDAC Inhibitors)	HIV-1 (Latent CD4⁺ T cells)	NKG2D (via epigenetic upregulation of MICA/B on target CD4⁺ T cells)	Reverses HIV-1 latency (“shock”); upregulates host stress ligands (MICA/B) on CD4⁺ T cells, unmasking latent reservoirs to render them highly susceptible to NKG2D-mediated NK cytotoxicity (“kill”).	Clinical Trials (e.g. chidamide + NK cells in NCT07577986)	[385, 386]
Chinese herbal medicines (CHMs)	*Panax ginseng*/Resveratrol	IAV, HBV	Intracellular MAPK (JNK/ERK1/2)	Directly upregulates perforin and granzyme B expression; enhances cytolytic cascades via IFN-γ-dependent pathways.	Pre-clinical/Traditional use	[392, 397, 398]
	*Astragalus membranaceus*/Artesunate	HBV	Immune microenvironment sensors	Reshapes the local hepatic milieu by drastically suppressing TGF-β1 and IL-10 production; rescues NK cells from functional exhaustion.	Pre-clinical	[119, 405, 406, 442–445]
	Ligustrazine hydrochloride (LHC)	Viral infections	CD3ε-ζ + isoform	Modulates surface receptor repertoire by suppressing specific isoforms; fine-tunes the balance between activating and inhibitory signals.	Pre-clinical	[407]

### Monoclonal antibodies

Disrupting inhibitory signaling cascades that constrain NK cell antiviral function is a core strategy for immunotherapeutic development, with monoclonal antibodies (mAbs) emerging as pivotal tools to reverse NK cell dysfunction [292]. Using mAbs to disrupt the binding of MHC-I molecules with inhibitory receptors to release inhibitory signals is a strategy to enhance anti-viral activities of NK cells [293, 294]. Monalizumab, an anti-NKG2A mAb blocking the CD94/NKG2A–HLA-E axis [295], successfully rescued NK-cell cytolysis and IFN-γ secretion in preclinical chronic HCV models [296]. However, although monalizumab has primarily progressed through Phase I/II/III clinical trials for advanced solid malignancies [297], robust clinical trials demonstrating its capacity to lower chronic HBV or HCV viral loads are still lacking. Similarly, lirilumab (anti-KIR mAb), which blocks the KIR2DL1/2/3–HLA-C inhibitory axis [298, 299], restored NK-cell-mediated cytolysis of HPV-infected cells in preclinical studies [298]. Nonetheless, Phase I/II clinical trials combining lirilumab with nivolumab have focused on tumor response in resectable head and neck cancers and have not yet demonstrated quantifiable reductions in the local HPV DNA viral load, highlighting a persistent gap between tumor regression and complete viral eradication [300].

Following their success in oncology, mAbs targeting immune checkpoints (PD-1, PD-L1, and CTLA-4) are under clinical translation to reverse the profound exhaustion of cytotoxic lymphocytes, including NK cells, during chronic viral infections [1, 3, 301]. In a Phase Ia/Ib clinical trial (NCT02443324) of virally suppressed chronic HBV patients without malignancy, a single dose of the anti-PD-1 mAb nivolumab safely induced significant HBsAg declines (>0.5 log_10_ IU/ml) in a subset of patients, with one patient achieving complete HBsAg clearance (functional cure) [302]. Similarly, the Phase Ib/IIa HBV002 trial evaluating the therapeutic vaccine VTP-300 combined with low-dose nivolumab demonstrated sustained HBsAg reductions exceeding 1.0 log_10_ IU/ml or complete clearance [303]. Translational evaluations confirm that PD-1 is pathologically upregulated on the CD56dim NK-cell subset during chronic HBV [55, 303]; thus, except for T cells, the clinical efficacy of these regimens may also be heavily driven by the functional rescue of exhausted NK-cell functions [55, 304]. Moreover, in non-oncology chronic HCV cohorts, a Phase I trial of nivolumab similarly showed restricted efficacy, with only three of 54 patients achieving a >4 log_10_ IU/ml reduction in HCV RNA [305] and a chronic HCV-HCC cohort treated with nivolumab alone achieved only transient viral load fluctuations without achieving sustained virologic response [306, 307]. However, combining nivolumab with ipilimumab (anti-CTLA-4) triggered a 9% HBV and 10% HCV virological breakthrough rate (defined as a ≥1 log_10_ viral load increase from baseline) [308], necessitating the combinatorial strategies, which have also been discussed in the text below.

In HIV-1 cure research, checkpoint inhibitors have been evaluated as potential latency-reversing agents (LRAs) under the “shock and kill” paradigm. In the Phase I CITN-12 clinical trial (NCT02595866) evaluating pembrolizumab (anti-PD-1) in people living with HIV (PLWH) and advanced cancers on stable ART, pembrolizumab successfully reversed HIV-1 latency (the “shock”), as evidenced by transient spikes in cell-associated unspliced HIV-1 RNA and low-level plasma viremia [309, 310]. However, pembrolizumab monotherapy failed to reduce the latent HIV-1 reservoir size (absolute HIV-1 DNA levels) [310, 311]. Correlative analyses revealed that while PD-1 blockade successfully unleashes NK cells by removing their inhibitory “brakes”, the reactivated latent cells can evade clearance by the rapid expression of viral accessory proteins (predominantly Nef and Vpu), which downregulate key activating and co-stimulatory ligands (such as MICA/B and CD48), rendering the HIV-1 reservoir virtually “invisible” to NK-cell cytotoxicity [259, 311–313].

Therapeutic mAbs that leverage ADCC represent another effective approach to activate NK cells [314]. For example, anti-CD30 mAbs engage CD16 (FcγRIIIa) on NK cells to eliminate CD30^+^ Hodgkin lymphoma cells in clinical trials, a mechanism that can be translatable to virus-driven lymphoproliferative diseases [315, 316]. Bispecific killer cell engagers (BiKEs) further enhance NK cell targeting specificity: anti-CD16-CD33 BiKEs override KIR-HLA I inhibitory signals in acute myeloid leukemia and myelodysplastic syndromes, and similar designs, which likewise link an anti-CD16 domain to virus-specific binding fragments (e.g. HIV gp160, HBV PreS1), are under preclinical evaluation to direct NK cells toward infected cells [317–320]. However, BiKEs lack intrinsic capacity to sustain NK cell proliferation and survival *in vivo*. To address this limitation, trispecific killer cell engagers (TriKEs) integrate IL-15 receptor-binding domains, which promote NK cell expansion, persistence, and antiviral efficacy, and findings supported by preclinical studies showing enhanced clearance of HIV-infected cells and HCMV reactivation [321–325]. Moreover, to counteract activation-induced CD16 shedding, emerging multi-specific killer engagers are being designed to co-engage CD16 with more stable activating receptors, such as NKG2C. The CD16-NKG2C-based tri-specific engagers not only bypass CD16 cleavage to maintain sustained immunological synapses but also drive the selective, antigen-targeted expansion of highly cytotoxic NKG2C^+^ adaptive NK cells [198].

However, the translational attempts above highlight several critical immunological concerns for the clinical applications of the anti-viral mAbs. In tissue-specific microenvironments highly enriched with NK cells, such as the liver where NK cells comprise up to 30%–50% of the lymphoid compartment [326, 327], checkpoint-mediated hyperactivation may trigger severe immunopathology and liver transaminase flares rather than coordinated viral clearance [328]. Besides, exhausted NK cells in chronic infections routinely co-express multiple, redundant inhibitory receptors (such as TIGIT, TIM-3, CTLA-4, and PD-1) [298], as exemplified by the limited antiviral efficacy of nivolumab alone against HCV [305] compared to the superior clearance outcomes achieved through its combination with ipilimumab [308]. Moreover, checkpoint inhibitors merely release the “brakes” [295]. If target cells have been stripped of activating ligands by viral accessory proteins (e.g. HCMV UL141 degrading CD112/CD155, or HIV-1 Nef downregulating MICA/B), the licensed NK cells will still lack the positive “trigger” required to initiate cytolytic granule exocytosis, rendering checkpoint blockade functionally inert [259, 329]. Therefore, single-agent therapies such as the anti-TIM-3 mAb sabatolimab, which showed preclinical promise [330], but saw its clinical development program terminated [331], necessitating combinatorial approaches [332]. For example, combined stimulation with IL-21 (upregulating NK cell surface expression of CD16, CD69, and NKG2D, enhancing IFN-γ production, cytolytic granule release, and cellular proliferation [333, 334]) and anti-TIGIT antibodies (relieving the IFN-γ production and inhibition of CD56^dim^ NK cells in chronic HBV infection [117, 332, 335]) partially reverses NK cell dysfunction in chronic HBV infection [332]. Therefore, the two act synergistically to accelerate HBsAg/HBeAg clearance by promoting IFN-γ secretion of splenic NK cells (rather than intrahepatic NK cells) in HBV-carrier mice, with IL-21 being an essential prerequisite for the anti-TIGIT-mediated HBV clearance effect [332].

Overall, these limitations underscore that effective anti-viral immunotherapy cannot solely rely on checkpoint blockade. Future strategies must move toward precision multiplexing, combining checkpoint inhibitors with therapeutic vaccines, cytokine adjuvants, or engineered bispecific engagers to simultaneously reverse NK-cell exhaustion and guarantee positive activating triggers for targeted viral clearance.

### Chimeric antigen receptor-modified NK cells

Chimeric antigen receptor (CAR)-modified immune cell therapy has revolutionized targeted immunotherapy, with CAR-T cells demonstrating remarkable efficacy in cancer treatment [336, 337]. Primary NK cell sources for preclinical and translational investigations predominantly include human peripheral blood mononuclear cells, umbilical cord blood mononuclear cells [338], immortalized NK cell lines (e.g. NK92) [339, 340], iPSCs [341, 342], and more recently, placenta-derived NK cells [343], each offering unique advantages in terms of accessibility, expandability, and clinical applicability [290, 338]. The clinical safety and applicability of these off-the-shelf sources have been recently validated in respiratory viral pandemics. For instance, the Phase I/II trial (NCT04365101) highlighted the safety of CYNK-001, a placenta-derived NK cell product, in treating severe COVID-19 [88]. Similarly, the Phase I RELEASE trial (NCT04900454) demonstrated the feasibility of infusing unmanipulated allogeneic NK cells into severe COVID-19 patients without exacerbating the systemic cytokine storm [344]. Building upon these foundational adoptive transfers, the design principle of CAR-NK cells aligns with NK cell activation mechanisms: by fusing a virus-specific single-chain variable fragment (scFv) to intracellular signaling domains, CAR-NK cells receive potent activating signals that override viral immune evasion [345–349]. Optimal CAR-NK design for antiviral applications uses NK cell-specific components, such as the DAP12 adaptor protein (critical for NKG2C/NKp44 signaling) [350–352] and costimulatory domains derived from NK cell activation receptors (e.g. 2B4, DNAM-1), which enhance signal transduction and cell persistence compared to T cell-derived costimulatory domains (e.g. CD28, 4-1BB) [353–356].

While early CAR-NK research focused on cancer, recent advancements have extended their application to viral infections, with targeted design against key viral antigens [352]. For SARS-CoV-2, CAR-NK cells engineered with scFv targeting the conserved S protein (e.g. S309-CAR-NK) or high-mannose glycosylation sites (H84T-BanLec-CAR-NK) effectively kill infected cells and suppress pseudovirus replication in preclinical studies [357–359]. Against HIV, universal CAR-NK cells recognizing multiple epitopes of gp160 via DNP-conjugated antibodies overcome viral diversity, enabling elimination of diverse HIV subtype-infected cells in primary cellular models [163, 360, 361]. For HBV, CAR-NK cells targeting HBsAg or PreS1 region (inspired by CAR-T studies) exhibit potent cytotoxicity against HBV-infected hepatocytes and reduce cccDNA levels in preclinical models [362–364]. Additionally, CAR-NK cells targeting EBV gp350 or latent membrane proteins have shown promise in preclinical models of EBV-related lymphoproliferative disorders [365, 366].

Despite the efficacy of targeting specific viral antigens, this approach faces the continuous threat of mutational escape (e.g. in Spike or HA glycoproteins) and sophisticated viral immune evasion mechanisms. Consequently, a paradigm shift is occurring toward exploiting conserved host-derived stress signals [3, 16]. Rather than engaging in an evolutionary arms race with hypermutable viruses, redirecting CAR-NK cells toward universally upregulated “induced-self” stress ligands (such as MICA/B) constructs a “theoretically mutation-resistant”, broad-spectrum antiviral platform [3, 16]. For example, in HIV-1 cure strategies, the clinical feasibility of adoptive NK cell therapy has been firmly established by recent landmark Phase I trials (NCT03899480 and NCT03346499), which demonstrated that haploidentical NK cells combined with the IL-15 superagonist N-803 (Anktiva) safely reduced HIV RNA-positive cells within lymphatic reservoirs [9, 367]. Building on these unmanipulated cellular platforms, combining NKG2D-directed CAR-NK cells with LRAs provides a synthetic bypass to overcome viral ligand downregulation, which lowers the activation threshold necessary to eradicate “shocked” CD4^+^ T cells presenting epigenetic stress markers [368, 369]. Furthermore, to counteract the sophisticated viral subversion where infected cells pathologically stabilize HLA-E to hijack the dominant NKG2A inhibitory checkpoint (as seen in HBV or HCMV), innovative “switch receptor” engineering (e.g. NKG2A/NKG2C chimeras) may rewire this viral shield into a therapeutic target. By converting the virus’s own evasion signal into a potent activating cascade, the design may effectively turn local immune tolerance into targeted viral clearance [3, 370]. Furthermore, engineering CARs with natural NK receptors (e.g. NKp46 or DNAM-1) enables therapeutic NK cells to broadly target residual host ligands (e.g. CD112/CD155) on infected cells, effectively restoring robust immunosurveillance against highly heterogeneous viral clones [1, 16].

Despite promising preclinical results above, antiviral CAR-NK therapy faces challenges, including limited *in vivo* persistence, viral immune evasion, and suboptimal tissue homing [289, 371]. Strategies to address these limitations include co-expression of cytokines (e.g. IL-15) to enhance persistence [372], dual-target CAR designs to avoid escape, and modification of chemokine receptors to improve homing to infected tissues [373, 374]. For instance, in a preclinical study, engineering CAR-NK cells to co-express the chemokine receptor CXCR5 effectively directs them into B-cell follicles—a major immunologically privileged sanctuary site for latent HIV-1 and EBV—whereas CXCR3 modification facilitates robust trafficking to the liver during chronic HBV or HCV infections [375]. Furthermore, to counteract the highly immunosuppressive microenvironments typical of chronic viral infections (which are frequently enriched in TGF-β and IL-10), developing “armored” CAR-NK cells equipped with dominant-negative TGF-β receptors (dnTGF-βR) represents a highly promising frontier. These sophisticated genetic augmentations not only shield the engineered NK cells from virus-induced exhaustion but also actively remodel the local immune milieu to reinvigorate endogenous antiviral immunosurveillance [368]. With ongoing optimization of CAR structures, such as fourth-generation “armored” CARs incorporating cytokine secretion, these engineered platforms are actively transitioning into human trials. Notably, the universal off-the-shelf NKG2D-ACE2 CAR-NK cells have entered Phase I/II clinical trials (NCT04324996) for severe COVID-19 [357]. This dual-targeting construct translates preclinical concepts into practice by utilizing an ACE2 decoy to neutralize circulating SARS-CoV-2 while engaging NKG2D to lyse infected host cells.

Beyond direct viral clearance, engineering CAR-NK cells to modulate the hyper-inflammatory microenvironment, such as the clinical “cytokine storm” observed in severe COVID-19 and influenza, represents a critical next-generation frontier [86, 357, 376]. To further adapt CAR-NK cells to hyper-inflammatory contexts, emerging bioengineering strategies can focus on “armored” or TRUCK-like designs. For instance, in a preclinical study, CAR-NK cells can be engineered to co-express soluble decoy receptors, such as soluble gp130-Fc (sgp130-Fc), to selectively neutralize pathogenic IL-6 trans-signaling and TNF-α complexes within the local microenvironment [377]. Simultaneously, utilizing membrane-bound cytokines (such as mbIL-15) rather than systemic administration ensures cell-autonomous survival and persistence while avoiding systemic hyper-inflammation [378]. These microenvironment-tailored designs could successfully transform CAR-NK cells from simple antiviral bullets into precise immunomodulators capable of resolving lethal immunopathology.

### Pharmacological modulation of NK cells

#### Small molecule compounds

While cellular engineering and mAbs represent advanced biologic approaches, small molecule compounds offer a highly scalable, cost-effective, and tissue-penetrable strategy to rejuvenate NK cell antiviral immunity [1, 2]. Rather than using broadly acting immunomodulators, current research focuses on targeted small molecules that can precisely reverse NK cell exhaustion or expose hidden viral reservoirs to NK cell attacks, some of which have now entered advanced preclinical evaluation or clinical trials [2, 379]. A major class of advancing compounds comprises Toll-like receptor 8 (TLR8) agonists [380]. Unlike traditional antivirals, TLR8 agonists act as innate immune pathway activators that remodel the immunosuppressive microenvironment [381]. A leading clinical-stage candidate is selgantolimod (GS-9688), an oral selective TLR8 agonist currently evaluated in Phase II clinical trials for chronic HBV infection [380, 381]. Clinical and *ex vivo* studies demonstrate that selgantolimod administration strongly stimulates hepatic mononuclear phagocytes to secrete IL-12 and IL-18 [381]. This localized cytokine release effectively breaks intrahepatic tolerance, inducing a profound activation of exhausted CD56^dim^ NK cells, restoring their IFN-γ production, and promoting targeted hepatocyte lysis [381].

Another breakthrough category involves Second Mitochondrial-derived Activator of Caspases (SMAC) analogs [382]. Originally developed as pro-apoptotic agents for oncology, SMAC analogs (also known as IAP antagonists) have demonstrated remarkable efficacy in clearing persistent viral infections [382]. For instance, APG-1387, a bivalent SMAC analog, has entered Phase I/II clinical trials for HBV-positive HCC and chronic HBV [382]. APG-1387 not only sensitizes HBV-infected cells to apoptosis but also significantly expands the NK cell population and enhances innate anti-tumor/antiviral immunity within the liver [382]. Concurrently, in HIV-1 cure research, the SMAC analog AZD5582 has emerged as a potent LRA that acts via the non-canonical NF-κB pathway [382]. Recent studies highlight that AZD5582 synergizes with NK cells by simultaneously reversing HIV-1 latency and lowering the activation threshold required for NK cells to recognize and eliminate the reactivated viral reservoir [368, 382]. As an inhibitor of apoptosis protein (IAP) antagonist, AZD5582 degrades cIAP1/2 and XIAP, which releases initiator caspase-8 and executioner caspase-3 from cellular inhibition within latently infected CD4^+^ T cells [383, 384]. This pharmacological blockade sensitizes the reactivated targets to extrinsic apoptosis mediated by death receptors (*e.g*. Fas or TRAIL-R) upon contact with cytolytic NK cells [383]. Consequently, this mechanism bypasses the apoptosis resistance and activating ligand downregulation engineered by the viral accessory protein Nef, enabling NK cells to efficiently eliminate the reactivated reservoir even under suboptimal receptor-ligand engagement [383, 384].

Moreover, in the HIV “shock and kill” paradigm, histone deacetylase (HDAC) inhibitors such as vorinostat and chidamide (CS055) do not only reactivate latent virus [385], but also actively upregulate host stress ligands (such as MICA/B) on reactivated CD4^+^ T cells, thereby making them highly vulnerable to NK cell-mediated targeting and clearance [385, 386]. This epigenetic modulation effectively unmasks the hidden viral reservoir, rendering the reactivated cells highly susceptible to natural cytotoxicity via the NKG2D receptor on endogenous or engineered NK cells [385, 386]. Clinical trials utilizing chidamide alongside NK cell immunotherapy are currently underway to translate this targeted clearance strategy into a functional cure for people living with HIV (e.g. NCT07577986).

Despite their promise, systemic administration of these potent small molecules carries risks of off-target toxicities, such as cytokine release syndrome [381, 387]. To bypass this limitation, future research should focus on targeted delivery systems such as nanoparticles to selectively concentrate these compounds within viral sanctuaries such as the liver or B-cell follicles [388]. Overall, combining localized small-molecule modulation with advanced NK cell therapies represents a safer and more effective strategy to achieve sustained viral clearance [1, 301]

#### Chinese herbal medicines

CHMs and their bioactive components have long been recognized for their immunomodulatory, antitumor, and antiviral properties, making them promising adjuvant immunomodulatory agents to synergistically enhance NK cell-mediated antiviral immunity [389, 390]. Unlike conventional antiviral drugs that directly target viral replication, CHMs act by regulating the host immune microenvironment, specifically promoting NK cell proliferation, enhancing cytotoxicity, and reversing functional exhaustion, thereby reinforcing the innate immune barrier against viral infections [391]. Several CHMs and their components have been validated for NK cell activation in preclinical antiviral models. For instance, the water extract of *Panax ginseng* [392, 393], sulfated polysaccharides isolated from *Lentinula edodes* (shiitake mushroom) and *Polyporus umbellatus* (zhuling) [394, 395], or *Angelica sinensis* (danggui) polysaccharides [396] all enhance NK cell functions against virus-infected cells via an IFN-γ-dependent pathway in IAV, HBV, or HCV models; resveratrol, a polyphenol abundant in polygonum cuspidatum and grapes, activates MAP kinases (JNK and ERK1/2) to increase perforin and granzyme B expression in NK cells, potentiating their cytotoxicity against IAV and HBV-infected cells [397–399]. G*anoderma lucidum* (lingzhi) triterpenoids enhance NK cell degranulation and ADCC activity by upregulating CD16 expression, a mechanism critical for targeting antibody-opsonized viruses (e.g. HIV, SARS-CoV-2) [400, 401]; notably, ω-3 polyunsaturated fatty acids (ω-3 PUFA), often integrated into traditional dietary therapies alongside CHMs, inhibit viral-induced inflammation and enhance NK cell antitumor/antiviral effector function by regulating lipid metabolism and reducing oxidative stress [402–404].

Another critical immunomodulatory effect of CHMs is the inhibition of immunosuppressive cytokines that impair NK cell function during chronic viral infections. *Astragalus membranaceus* (huangqi), a widely used CHM, significantly reduces the secretion of TGF-β1, thereby restoring NK cell cytotoxicity against HBV-infected cells [119, 405]. Artesunate, a derivative of *Artemisia annua* (qinghao), inhibits IL-10 production and reverses NK cell dysfunction, synergizing with direct-acting antivirals to accelerate viral clearance [406]. Additionally, LHC derived from *Ligusticum wallichii* (chuanxiong) modulates NK cell receptor expression by suppressing CD3ε-ζ^+^ isoform levels, enhancing the balance between activating and inhibitory signals [407].

Despite compelling preclinical evidence, the clinical translation of CHMs for enhancing NK cell-mediated antiviral therapy faces challenges, including inconsistent bioactive component purity, unclear optimal dosing, and limited large-scale clinical trials [408, 409]. Future research should focus on isolating and standardizing key bioactive compounds, elucidating their precise molecular targets, and evaluating their synergistic effects with conventional antiviral therapies in clinical settings. With systematic optimization, CHMs hold great potential as safe, cost-effective adjuvants to boost NK cell function and improve outcomes of viral infections, particularly chronic infections where NK cell exhaustion is a hallmark.

## Next-generation NK cell antiviral therapies

NK cells are integral to antiviral defense, capable of direct cytotoxicity, immunomodulation, and developing adaptive-like features [11]. However, the successful clinical translation of NK cell-based antiviral immunotherapies remains impeded by several critical biological limitations. Unlike T cells, unmanipulated NK cells exhibit a remarkably short lifespan *in vivo* and rely heavily on continuous cytokine support (such as IL-2 or IL-15) for sustained survival and expansion, which frequently restricts long-term viral containment [106]. This limited persistence is further compounded by inefficient tissue homing and trafficking, as systemically infused NK cells often lack the specific chemokine receptors required to effectively infiltrate restrictive viral sanctuaries, such as the central nervous system or lymph node follicles, thereby permitting ongoing cryptic replication [11]. Upon reaching target tissues, NK cells must also navigate sophisticated viral immune evasion mechanisms, where persistent pathogens remodel the local microenvironment by upregulating inhibitory checkpoints, shedding activating ligands, and secreting immunosuppressive cytokines to drive functional exhaustion [3]. Furthermore, therapeutic outcomes across patient cohorts are often unpredictable due to significant inter-individual heterogeneity, which is shaped by polymorphic KIR haplotypes, host HLA backgrounds, and immunological imprinting from prior environmental exposures such as chronic cytomegalovirus infection [410]. Therefore, translating NK cell biology into effective, standardized therapies requires next-generation cellular engineering and precision medicine strategies specifically designed to overcome these key translational hurdles.

A primary translational focus must be the engineering of next-generation NK cell products with enhanced survival, homing, and resistance to viral evasion. This requires genetic modifications to promote *in vivo* persistence, such as the expression of cytokines like IL-15, and the tailoring of homing receptors (e.g. CXCR3, CCR5) to direct effector cells to sites of infection or latency [411]. Furthermore, arming NK cells, particularly CAR-NK cells, with mechanisms to counteract immunosuppressive pathways (e.g. dominant-negative TGF-β receptors) or to simultaneously engage multiple activating signals can render them more effective in the hostile microenvironment of chronic infection [412, 413]. Given the redundancy of viral immune evasion, combinatorial therapeutic paradigms will be essential. The synergy between checkpoint blockade (e.g. anti-NKG2A, anti-TIGIT) and bispecific engagers (BiKEs/TriKEs) or adoptive NK cell transfer offers a promising avenue to overcome inhibition and restore cytotoxicity against viruses like HCMV and HIV [414].

Concurrently, the adaptive and antigen-specific NK cells open novel avenues for vaccine design and personalized immunotherapy. Deciphering the molecular basis of antigen recognition in human NK cells is critical to rationally design vaccines that prime durable NK cell memory [415]. Under a precision medicine framework, using genetic and cellular biomarkers to match donors and recipients is essential to maximize the efficacy of NK cell therapies. In this context, KIR/HLA genotyping serves as a vital genetic stratification tool. Matching or strategically mismatching donor inhibitory KIR receptors with recipient HLA class I ligands directly influences the functional licensing and activation threshold of the infused cells [1]. For example, selecting donors with favorable activating KIR profiles, such as the KIR B haplotype or the KIR3DS1 gene, is strongly linked to superior clinical control over viral infections [1]. In addition to these genetic traits, CMV-driven immune imprinting acts as a powerful cellular biomarker. Prior exposure of a donor to cytomegalovirus permanently reshapes their NK cell pool, expanding highly cytolytic NKG2C^+^CD57^+^ FcϵRIγ^−^ adaptive NK cells [201, 207]. Choosing CMV-seropositive donors ensures the transfer of these pre-expanded cells, which possess enhanced ADCC and resist tissue-level immunosuppression, thereby predicting better outcomes in patients suffering from refractory viral reactivation [51, 201]. Integrating these genetic and environmental biomarkers allows clinicians to design personalized, highly targeted NK cell therapies instead of a one-size-fits-all approach [16].

Beyond direct anti-viral applications, the antigen-specific memory NK cells described in this review (e.g. HCMV-induced NKG2C^+^ or IAV-NP-specific subsets) offer an innovative paradigm for cancer immunotherapy. A promising translational strategy involves “re-labeling” tumor cells with specific viral antigens to harness pre-existing anti-viral innate memory. By delivering viral nonameric peptides (such as HCMV UL40 or IAV NP-derived peptides) into the tumor microenvironment (TME) via oncolytic viruses or nanoparticle systems, tumor cells can be forced to present these “memory triggers” through HLA-E molecules [42, 43]. This strategy, inducing “viral mimicry” in the TME, can effectively recruit and activate the highly cytotoxic memory NK cells already present in a large proportion of the population [416]. Unlike traditional CAR-NK therapies that require complex cell engineering, this “viral mimicry” strategy leverages the host’s endogenous adaptive NK cell pool to bypass the immunosuppressive TME and initiate a robust anti-tumor response. Future research should focus on optimizing the delivery efficiency of these viral peptides and evaluating the synergy between this memory-driven approach and existing immune checkpoint inhibitors [42, 43, 416]. Besides, realizing the “off-the-shelf” potential of NK cell therapies demands solutions to manufacturing and logistical challenges. The standardization of NK cell derivation from sources such as like iPSCs and the optimization of cryopreservation protocols are pivotal steps toward scalable, readily available products capable of responding to emerging viral threats [417, 418].

With their modularity, scalability, and robust immunogenicity, messenger RNA (mRNA) technologies have transformed prophylaxis and immunotherapy [419]. Currently, a key translational challenge lies in optimizing these genetic platforms to mobilize both innate and adaptive immunity [419]. Prophylactic trials, such as longitudinal cohorts of BNT162b2-vaccinated individuals, show that baseline NK-cell frequencies and activation kinetics directly correlate with vaccine-induced antibody titers, positioning NK cells as crucial biomarkers for vaccine responsiveness [420]. For therapeutic applications, mRNA vaccines show robust synergism with adoptive NK-cell therapies. For example, in EBV-associated nasopharyngeal carcinoma, combining a polyepitope mRNA vaccine with adoptive cytotoxic NK cells led to tumor eradication in humanized mice, significantly improving immune infiltration into the TME [421]. Next-generation designs are further optimized by co-delivering viral antigens alongside NK-cell-stimulating cytokines (such as IL-15/IL-15Rα complexes) within the same open reading frame to bypass viral MHC-I downregulation [422]. This can be further enhanced by utilizing circular mRNA (circRNA) to extend *in vivo* translation and prime memory NK cells [423], or self-amplifying mRNA (samRNA) to generate intracellular double-stranded RNA intermediates that trigger type I interferons and activate NK-cell degranulation [424]. Simultaneously, transfecting expanded NK cells with CAR-encoding mRNA-LNPs achieves up to 95% transfection efficiency, producing transient, highly cytotoxic CAR-NK cells while avoiding the insertional mutagenesis risks of viral vectors [424]. Looking forward, future clinical strategies should leverage these advanced platforms to design multi-targeted mRNA vaccines that simultaneously prime adaptive T cells and memory NK cells to prevent viral mutational escape. Furthermore, establishing standardized, clinic-ready combinations of personalized therapeutic mRNA vaccines and off-the-shelf, non-viral CAR-NK cells represents a promising frontier to achieve substantial clearance of refractory viral reservoirs.

In summary, the next era of NK cell-based antiviral therapy will be defined by integrated approaches that combine advanced cell engineering, intelligent drug combinations, and biomarker-guided clinical application to transform these innate immune sentinels into powerful and precise clinical tools.
